# Post-discharge evaluation of patients with hospital-acquired pressure injuries: prospective cohort study^*^

**DOI:** 10.1590/1518-8345.7866.4768

**Published:** 2026-06-15

**Authors:** Michelle Venâncio Hong, Bruna Cristina Velozo, Isabela Novello, Hélio Amante Miot, Meire Cristina Novelli e Castro, Luciana Patrícia Fernandes Abbade

**Affiliations:** 1 Universidade Estadual Paulista Júlio de Mesquita Filho, Faculdade de Medicina de Botucatu, Departamento de Enfermagem, Botucatu, SP, Brazil.; 2 Universidade Estadual Paulista Júlio de Mesquita Filho, Faculdade de Medicina de Botucatu, Departamento de Dermatologia, Botucatu, SP, Brazil.

**Keywords:** Pressure Ulcer, Patient Discharge, Home Care Services, Home Nursing, Continuity of Patient Care, Wound Healing.

## Abstract

**Objective:**

to evaluate the healing evolution of pressure injuries developed in the hospital setting among adult patients in the post-discharge period, as well as the complications and care provided to these patients.

**Method:**

prospective cohort study with patients who developed pressure injuries during hospitalization and were subsequently discharged. Data were collected at 7, 15, 30, and 60 days after hospital discharge.

**Results:**

the sample included 113 patients, 63 men (55.8%), with a mean age of 64.6 years old (± 15.1). A total of 246 injuries occurred, predominantly in the sacral region (91; 37.0%) and classified as stage 2 (117; 47.6%). Readmission rates ranged from 10.6% to 16.8% over 60 days and 25 deaths (22.1%) occurred. At the last interview, 57 patients participated, of whom 29 (50.9%) remained bedridden or with very limited mobility, while 48 (84.2%) showed improvement or complete healing. Stroke occurred in 22 (19.5%) patients and was an unfavorable factor in the healing process (HR = 0.6; p = 0.021).

**Conclusion:**

during the 60 days after discharge, patients experienced unfavorable outcomes, including death, hospital readmission, and non-healing of pressure injuries. Stroke was associated with a slower progression of the healing process.

## Highlights

**(1)** The mortality rate after hospital discharge was 22%. **(2)** Readmission rates ranged from 10.6% to 16.8%. **(3)** Stroke as a comorbidity adversely affected the healing process. **(4)** At 60 days after discharge, 50.9% of pressure injuries had improved and 33.3% were fully healed. **(5)** After discharge, 50.6% of patients did not receive care in the first week.

## Introduction

Pressure injury (PI) is characterized by damage to the skin and/or underlying tissues caused by external force applied to the skin in areas of bony prominence or through the use of medical devices or other objects. Common in hospitals, PI is considered an adverse event and serves as an indicator of the quality of care provided in health services^1^.

Despite advances in prevention strategies, the incidence of PI has remained relatively stable in recent years^2^. Globally, the prevalence of PI is estimated at 12.8% and the incidence of hospital-acquired PI in adults at 8.5%^3^. In Brazil, more than 60,000 cases were reported in 2023^4^. There are significant variations in incidence across hospital sectors, with Intensive Care Units (ICU) showing the highest prevalence rates^5^ ^-^ ^6^.

PI may occur due to patient-related factors such as advanced age, skin fragility^7^, reduced mobility, shear, prolonged hospitalizations^8^ and malnutrition, which is a relevant risk factor^9^. In addition to being a constant challenge in health care, PI is one of the most difficult clinical issues to manage. It negatively affects patients emotionally, mentally, physically and socially, resulting in an overall reduction in quality of life^10^. 

PI often increases dependence on health systems and support networks^10^, leading to a higher demand for care both during hospitalization and during the transition of care at discharge. At discharge, patients and their families face vulnerabilities related to safety, often due to inconsistencies in essential information for healing, such as topical care, pressure relief measures and frequent skin assessments^11^.

Environmental changes resulting from hospitalization affect not only the patient but also their relatives or caregivers. Returning home with a PI requires not only adjustments to daily routines but also the addition of new responsibilities within the household^12^. The use of supportive objects, appropriate furniture^13^ and an organized physical environment for hygiene and safe mobility, along with fall-prevention measures, are recommended^14^. These modifications, combined with caregiver support, help ensure effective and safe home care. However, these aspects are often not discussed during hospitalization and discharge, which may compromise patient care and hinder the healing process^15^.

It is known that proper guidance for family members and caregivers is essential for both the treatment and prevention of PI. In this regard, the standardization of post-discharge guidelines helps to mitigate gaps in knowledge on the subject^16^. Furthermore, the care of patients with PI is highly complex, especially for lay caregivers, and may lead to unfavorable outcomes such as hospital readmissions, complications, and reduced quality of life^15^. 

Despite the relevance of this topic, there is a lack of studies addressing postdischarge outcomes of patients with health service-acquired PI and the ways in which family members or informal caregivers manage these injuries at home. Thus, it is important to conduct research on these outcomes in patients discharged from hospital, as well as to investigate their evolutionary characteristics in the extra-hospital setting.

Therefore, the aim of this study was to evaluate the healing evolution of PI developed in the hospital setting among adult patients in the post-discharge period, as well as the complications and care provided to these patients.

## Method

### Type of study

This is a prospective cohort study, reported according to the Strengthening the Reporting of Observational studies in Epidemiology (STROBE) guidelines^17^.

### Study participants and location

The study included adult patients who developed PI during hospitalization in wards or Intensive Care Units (ICUs) of two public teaching hospitals located in the city of Botucatu, in the countryside of São Paulo, Brazil. Both hospitals are under the same administration: one is a secondary-level hospital, and the other is tertiary-level. 

The institutions share the same Wound Care Committee, composed of specialized nurses who provide care in both hospitals to patients with PI or other wounds requiring specific treatment. Patients with PI, monitored during hospitalization and subsequently discharged, are guided by the Wound Care Committee and bedside nurses to ensure a safe transition at discharge. A counter-referral letter is provided for follow-up in Primary Care, including instructions and recommendations regarding the dressings performed. 

For this study, patients evaluated by the Wound Care Committee and discharged between January 2022 and January 2023 were included. 

### Eligibility criteria

The inclusion criteria were: patients of both genders who developed hospital-acquired PI, aged over 18 years old, evaluated by the Wound Care Committee of the institutions during hospitalization and discharged with PI.

Patients who already presented PI when admitted to the hospital, even if new lesions developed during hospitalization and patients with PI who were transferred to institutions not included in the study.

### Outcomes

The primary outcome was the evaluation of factors associated with the progression of the healing process within 60 days after hospital discharge. Based on caregivers’ perception, participants were divided into two groups: favorable evolution of the healing process (total healing or improvement) and unfavorable evolution of the healing process (stable or worsening). The following criteria were used in the interviews conducted with patients, family members, or caregivers: (i) Signs of improvement: presence of reddish tissue (granulation tissue), reduced exudate, and decreased wound area; and (ii) Signs of worsening: increased necrosis, odor, pain, exudate, and an unchanged or enlarged wound area.

The secondary outcomes were: (i) to describe the baseline sociodemographic, clinical and therapeutic characteristics of patients who developed PI in the hospital setting; (ii) to report complications (death and readmission) and variables related to the care of patients during the post-discharge follow-up period, obtained through interviews.

### Data collection instruments 

For the active search of patients, reports from the Wound Care Committee in the hospital system were reviewed. These reports identified whether the PI was community-acquired or hospital-acquired and all patients who developed hospital-acquired PI were selected. 

To verify compliance with the eligibility criteria, patient information was obtained from electronic medical records. After confirmation and consent to participate, documented through the signing of the Informed Consent Form (ICF), telephone interviews were conducted with patients, family members, or caregivers. During the interviews, information was collected regarding discharge instructions and adherence, type of dressing used, access to health services, general clinical conditions, and the healing process of PI.

### Data collection and study variables

Data collection covered both the hospitalization period (variables obtained from electronic medical records) and post-discharge follow-up at days 7 (D7), 15 (D15), 30 (D30), and 60 (D60), with variables obtained through interviews. The maximum follow-up period of 60 days was established considering the feasibility of obtaining the necessary information within this interval to assess out-of-hospital care and the progression of the healing process. This definition was based on a retrospective cohort study that identified a mean healing time of 46 days for PI^18^. 

If the participant or caregiver did not answer the phone after three attempts, the interview for that day was considered missed. However, the participant remained in the study and was included in subsequent interviews until completion. As this was an observational study, there was no interference in patients’ care routines or discharge instructions.

Data were collected using an online form (Google Forms^®^) developed by the researchers and validated after a pilot test with ten patients. The final form included dependent and independent study variables, such as:

- Sociodemographic data: sex, age, self-declared race/ethnicity (White, Black, Brown, Yellow, and/or other) and family income.
- Clinical variables: relevant comorbidities, length of hospitalization (in days), patient’s origin before hospitalization, reason for hospitalization, complications during hospitalization and time to Wound Care Committee evaluation for PI (in days).
- Number of PI developed per patient, anatomical location and classification.
- Therapeutic approach to PI in the hospital setting

- Post-discharge interview variables included: complications (death and readmission); patient’s place of residence after discharge [own home, relatives’/friends’ home or Long-Term Care Institutions for Older Adults (LTCF)]; type of caregiver (family member, friend, nursing technician/assistant or trained caregiver without formal education); whether guidance was provided after hospital discharge; adherence to post-discharge instructions (asked whether adherence was complete, partial or none); health services accessed after discharge; type and frequency of care; patient mobility at the time of the interview according to the “mobility” dimension of the Braden Scale^(19)^; measures adopted for post-discharge treatment of PI; type of dressing used at the time of the interview and post-discharge progression of the healing process, as reported by the patient/caregiver (complete healing, improvement, stabilization or worsening).

### Definition of the sample

According to previous data from the Wound Care Committee of the hospitals included in the study, 269 hospital-acquired PI were recorded between October 2019 and October 2020. The sample size for this study was determined by convenience, considering data collection over a 12-month period for participant inclusion.

### Data analysis

Descriptive statistics were applied, using frequency and percentage for categorical variables and mean, standard deviation, median, minimum, and maximum for quantitative variables. The normality of quantitative (continuous) variable distributions was assessed using the Shapiro-Wilk test. Results were presented as means (± standard deviation) for normally distributed variables and as medians (p25-p75) for non-normally distributed variables^20^.

For analysis, participants were divided into two groups: those with favorable evolution (complete healing or improvement of PI) and those with unfavorable evolution (stable or worsening PI). Survival curve analysis (Kaplan-Meier)^21^ using the Log Rank test (Mantel-Cox) was performed to identify factors influencing favorable evolution (improvement/healing). In cases with differences in survival, the effect size was estimated by the Hazard Ratio (HR) and its 95% confidence interval (95% CI)^22^. Cases that did not reach the outcome of favorable evolution were censored, as they left the study due to loss to follow-up (death, readmission, or non-completion of the interview).

The variables analyzed were chosen according to their possible influence on the healing process and dichotomized according to their average frequencies, such as gender, age over 65 years, White versus non-White race/ethnicity, hospitalization period of 30 days, minimum time of 15 days for evaluation by the Wound Care Committee, number of up to two PI and comorbidities [arterial hypertension (AH), diabetes mellitus (DM) and stroke (CVA, Cerebrovascular Accident)].

All statistical analyses were performed using SPSS 23.0 (IBM). A 95% confidence interval (CI) and a significance level of 5% (p < 0.05) were considered.

### Ethical aspects

This study was approved by the Research Ethics Committee and recognized by the National Research Ethics Commission under Certificate of Presentation for Ethical Consideration No. 53331521.6.0000.5411, in compliance with Resolutions 466/2012 and 510/2016 of the National Health Council. After clarification and explanation regarding the study, participation required prior consent from the patient and/or their closest legal guardian. When the patient was no longer in the institution due to hospital discharge, the ICF was obtained remotely. 

## Results

During the study period, the hospital system recorded 962 evaluations with a diagnosis of “hospital-acquired pressure injury,” conducted by the Wound Care Committee. After removing duplicates, that is, patients readmitted at different times, a total of 461 patients remained. Those who did not meet the eligibility criteria were excluded, that is, patients who died during hospitalization (n = 248), patients under 18 years of age (n = 27), those transferred to another hospital unit (n = 9), those who presented PI at the time of hospital admission or another type of injury (n = 35) and those for whom a consultation with the Wound Care Committee was not requested (n = 9). Therefore, 133 patients were eligible, but 113 consented to participate and were included in the study.

### Sociodemographic, clinical, and therapeutic characteristics of patients who developed hospital-acquired pressure injuries

Table 1 presents the sociodemographic and clinical data of the 113 participants. The majority were male (63; 55.8%), with a mean age of 64.6 (± 15.1) years old, self-declared White race/ethnicity (85; 75.2%) and a reported family income of one to three minimum wages (49; 43.3%). The main comorbidity was AH (74; 65.5%). Length of hospitalization ranged from 7 to 148 days, with a median of 40.0 (22.0 - 57.0). Before hospitalization, 63 (55.8%) were living in their own homes and the main reasons for admission were injuries due to external causes (25; 22.1%) and diseases of the nervous system (24; 21.2%). Infections (52; 46.0%) were the most frequently observed complications. 

The Wound Care Committee was called after a median of 13.0 (6.0 - 29.0) days of hospitalization. A total of 246 PIs were identified in 113 patients, most of whom presented with only one lesion (58; 51.3%). The sacral region (91; 37.0%) and heel (49; 19.9%) were the most frequently affected anatomical sites, and stage 2 (117; 47.6%) was the most common classification (Table 2).

**Table 1:** Sociodemographic and clinical data of participants with hospital-acquired pressure injuries (n = 113). Botucatu, SP, Brazil, 2022-2023

**Variables**	**n (%)**
**Sex**		
Male	63 (55.8)
Female	50 (44.2)
**Age in years old**		
Minimum and maximum	22- 95
Mean (± standard deviation)	64.6 (15.1)
**Skin color/Race**		
White	85 (72.2)
Black	17 (15.0)
Brown	11 (9.7)
**Family income**		
Less than 1 MW*	9 (7.9)
1 to 3 MW*	49 (43.3)
4 to 10 MW*	21 (18.5)
Not reported	34 (30)
**Comorbidities^†^**		
Arterial hypertension	74 (65.5)
Diabetes mellitus	34 (30.1)
Stroke	22 (19.5)
**Length of hospitalization in days**		
Minimum and maximum	7 to 148
Median (p25 - p75)	40.0 (22.0 - 57.0)
**Origin before hospitalization**		
Own residence	74 (65.5)
Transferred from another hospitalization	32 (28.3)
Long-Term Care Institutions for Older Adults	5 (4.4)
No answer	2 (1.8)
**Reason for hospitalization^†^**		
Injuries due to external causes^‡^	25 (22.1)
Disease of the nervous system	24 (21.2)
Disease of the respiratory system	21 (18.6)
Disease of the circulatory system	19 (16.8)
Neoplasms	11 (9.7)
Endocrine, metabolic, and nutritional diseases	10 (8.8)
Disease of the genitourinary system	10 (8.8)
Disease of the digestive system	8 (7.1)
Autoimmune disease	2 (1.8)
**Complications during hospitalization^†^**		
Infections	52 (46.0)
Respiratory complications	43 (38.1)
Cardiorespiratory arrest	10 (8.8)
Cardiovascular complications	10 (8.8)
Positive COVID-19 test	10 (8.8)
Deep vein thrombosis	3 (2.7)
Stroke	2 (1.8)
Acute myocardial infarction	1 (0.9)
No complications	10 (8.8)

*MW = Minimum wage in force in 2022 (BRL 1,212.00), Brazil; ^†^More than one result per patient; ^‡^Injuries due to external causes: fractures, falls, assaults, and transport-related accidents

**Table 2:** Characteristics of the 246 hospital-acquired pressure injuries (n = 113). Botucatu, SP, Brazil, 2022-2023

**Variables**	**n (%)**
**Patient-related**	**n = 113**
**Length of hospitalization (in days) until the Wound Care Committee was called**		
Minimum and maximum	1 - 105
Median (p25 - p75)	13.0 (6.0 - 29.0)
**Number of pressure injuries developed per patient**		
One	58 (51.3)
Three	19 (16.8)
More than three	19 (16.8)
Two	17 (15.0)
**Pressure injury-related**	**n = 246**
**Anatomical location**	
Sacral	91 (37.0)
Calcaneus	49 (19.9)
Ischial	21 *(*8.5)
Ankle	17 (6.9)
Auricle	10 (4.1)
Trochanter	10 (4.1)
Feet or hands	8 (3.3)
Occipital	8 (3.3)
Other anatomical regions*	32 (14.1)
**Classification of pressure injuries**		
Stage 2	117 (47.6)
Stage 1	60 (24.4)
Unstageable injury	31 (12.6)
Stage 3	12 (4.9)
Deep tissue injury	11 (4.5)
Medical device-related injury	11 (4.5)
Stage 4	4 (1.6)

*Other anatomical regions: lateral or medial malleolus, spine, scapula, tibia, nose, knee, lips, and elbow

Table 3 shows the therapeutic approaches and pressure relief measures adopted. It is identified that the most prescribed topical treatment was polyhexamethylene biguanide in liquid and/or gel form (109; 44.3%). The most recommended preventive measure was repositioning in bed every two hours (173; 70.3%). It is noteworthy that the air mattress was indicated for only 38 participants (15.4%).

**Table 3:** Topical therapeutic approach and pressure relief guidance for 113 patients with 246 hospital-acquired pressure injuries. Botucatu, SP, Brazil, 2022-2023

**Variables**	**n (%)**
Topical treatment in the hospital setting*		
Polyhexamethylene biguanide (PHMB) liquid or gel	109 (44.3)
Hydrogel	65 (26.4)
Skin protectants^†^	89 (36.2)
Papain	21 (8.5)
Calcium alginate dressing	12 (4.9)
Polyurethane/hydro polymer foam dressing	9 (3.7)
Collagenase	8 (3.3)
Essential fatty acids	6 (2.4)
Silver-containing hydrofiber	6 (2.4)
Hydrocolloid	3 (1.2)
Silver sulfadiazine	2 (0.8)
Negative pressure dressing	1 (0.4)
Guidance from the Wound Care Committee*		
Bed repositioning every two hours	77 (68.1)
Cushions/pillows	30 (26.5)
Skin hydration and/or preventive measures	29 (25.6)
Pyramidal mattress	27 (23.9)
Daily dressing change	23 (20.3)
Heel offloading	17 (15.0)
Skin protectants^†^	17 (35.4)
Air mattress	14 (12.4)
No guidance	2 (1.7)

*Each patient could have received more than one treatment or recommendation during hospitalization; ^†^Skin protectants (barrier cream, spray film, and/or polyurethane film)

### Results of the post-discharge follow-up period

The variables from the interviews conducted during the 60-day post-discharge period are presented in Table 4. A total of 25/113 (22.1%) deaths were recorded during this period. Readmission numbers varied across interviews: in the first week after discharge, 19 (16.8%) patients returned to the hospital, while at D60, 12 (10.6%) were readmitted. Throughout the interviews, the number of patients/caregivers decreased due to complications (death, readmission or loss to follow-up from unanswered phone calls). Initially, 79 participants (69.9%) were interviewed at D7, but by D60, only 57 (50.4%) remained from the total sample.

**Table 4:** Variables related to the 113 patients with hospital-acquired pressure injuries during the 60-day post-discharge period. Botucatu, SP, Brazil, 2022-2023

**Variables**	D7* n (%)	D15* n (%)	D30* n (%)	D60* n (%)
**Not interviewed - Reasons**	34 (30)	38 (33.6)	36 (31.8)	36 (35.4)
Death^†^	5 (4.4)	10 (8.8)	15 (13.3)	25 (22.1)
Readmission	19 (16.8)	17 (15.0)	19 (16.8)	12 (10.6)
Did not answer telephone contact	10 (8.8)	16 (14.2)	12 (10.6)	19 (16.8)
**Interviewed**	79 (69.9)	70 (61.9)	67 (59.3)	57 (50.4)
**Place of stay**				
Own residence	46 (58.2)	41 (58.6)	43 (64.2)	36 (63.2)
Family and/or friends’ residence	28 (35.4)	23 (32.9)	19 (28.4)	18 (31.6)
Long-Term Care Institutions for Older Adults	5 (6.3)	6 (8.6)	5 (7.5)	3 (5.3)
**Caregivers^‡^**				
Family member	74 (75.5)	64 (68.8)	61 (70.9)	51 (89.5)
Caregiver without formal education	13 (13.3)	16 (17.2)	16 (18.6)	8 (14.0)
Nursing technician/assistant	9 (9.2)	11 (11.8)	8 (9.3)	4 (7.0)
Friend	2 (2.0)	2 (2.2)	1 (1.2)	1 (1.8)
**Received guidance on pressure injuries**				
Yes	61 (77.2)	62 (88.6)	55 (82.1)	49 (86.0)
No	18 (22.8)	8 (11.4)	12 (17.9)	8 (14.0)
**Adherence to guidance on pressure injuries**				
Yes, completely	57 (72.2)	59 (84.3)	54 (80.6)	48 (84.2)
Yes, partially	4 (5.1)	4 (5.7)	1 (1.5)	0 (0.0)
No	18 (22.8)	7 (10.0)	12 (17.9)	9 (15.8)
**Care assistance^‡^**				
No assistance	40 (50.6)	23 (32.9)	23 (34.3)	20 (35.1)
Primary Health Care	29 (36.7)	28 (40.0)	20 (29.9)	19 (33.3)
Specialized Outpatient Clinic and/or Private Clinic	7 (8.9)	23 (32.9)	25 (37.3)	19 (33.3)
Emergency Department	4 (5.1)	NA	1 (1.5)	1 (1.8)
**Type of care^‡^**				
No assistance	40 (50.6)	23 (32.9)	23 (34.3)	20 (35.1)
Home visit	30 (38.0)	29 (41.4)	24 (35.8)	20 (35.1)
Care provided at the health facility itself	9 (11.4)	19 (27.1)	22 (32.8)	18 (31.6)
**Frequency of care**				
No assistance	40 (50.6)	23 (32.9)	23 (34.3)	20 (35.1)
Once a week	32 (40.5)	26 (37.1)	21 (31.3)	16 (28.1)
Biweekly	-	21 (30.0)	15 (22.4)	5 (8.8)
Monthly	-	-	10 (14.9)	16 (28.1)
**Mobility**				
Bedridden	40 (50.6)	26 (37.1)	23 (34.3)	16 (28.1)
Very limited	25 (31.6)	28 (40.0)	25 (37.3)	13 (22.8)
Mild limitation	9 (11.4)	13 (18.6)	11 (16.4)	18 (31.6)
No limitation	5 (6.3)	3 (4.3)	8 (11.9)	10 (17.5)
**Pressure relief measures^‡^**				
Repositioning	77 (97.5)	68 (97.1)	61 (91.0)	46 (80.7)
Cushions	31 (39.2)	30 (42.9)	29 (43.3)	24 (42.1)
Pyramidal and air mattresses	17 (21.5)	23 (32.9)	21 (31.3)	10 (17.5)
**Dressing used^‡^**				
Barrier cream	37 (46.8)	28 (40.0)	28 (41.8)	18 (31.6)
Hydrogels/papain	37 (46.8)	34 (48.6)	41 (58.6)	20 (35.1)
Essential fatty acid	26 (32.9)	24 (34.3)	19 (28.4)	14 (24.6)
No product used	3 (3.8)	4 (5.7)	8 (11.9)	13 (22.8)
Others^§^	12 (15.2)	14 (20.0)	7 (10.4)	14 (24.6)
**Evolution of the healing process**				
Improvement	53 (67.1)	54 (77.1)	49 (73.1)	29 (50.9)
Stabilization	20 (25.3)	8 (11.4)	3 (4.5)	6 (10.5)
Total healing	5 (6.3)	6 (8.6)	11 (16.4)	19 (33.3)
Worsening	1 (1.3)	2 (2.9)	4 (6.0)	3 (5.3)

*D7, D15, D30 and D60: They refer to the follow-up days after hospital discharge, namely the 7th, 15th, 30th, and 60th day; ^†^Death: from D15 onward, the total number of deaths represented the cumulative sum of events that occurred in previous visit; ^‡^More than one result per patient; ^§^Other dressings: spray film, collagenase, calcium and sodium alginate dressing, and polyhexamethylene biguanide (PHMB) in liquid or gel form

The patients own residence was the post-discharge place of stay for most participants. Patients’ caregivers were their own relatives [74/79 at D7 (75.5%); 51/57 at D60 (89.4%)]. Regarding guidance on PI, the first interview (D7) used the time of hospital discharge as the reference point. Of the interviewees, 61/79 (77.2%) reported having received guidance in the hospital.

It is noteworthy that Primary Health Care was the service that most provided care at D7, with 29/79 of the cases (36.7%). There was a proportion of patients without care related to PI management from the first interviews, with 40/79 (50.6%) at D7 and 20/57 (35.1%) patients at D60.

As for mobility, at D7, 40/79 (50.6%) patients were classified as bedridden. However, there was progressive improvement in mobility, with the classifications “mild limitation” and “no limitation” increasing by 50% in the last interview. Nevertheless, at D60, 29/57 (50.9%) were still bedridden or with very limited mobility. The most frequently adopted pressure relief measure was repositioning, in 77/79 (97.5%) patients at D7 and in 46/57 (80.7%) patients at D60. Hydrogel and papain were the most used dressings, in 37/79 (46.8%) at D7. 

According to reports from patients and/or caregivers, at D60, improvement or healing of the PI was observed in 48/57 patients (84.2%). 

### Factors related to the evolution of the healing process of pressure injuries

To identify which factors significantly influenced the healing process, the responses from the last interview (D60) regarding PIs outcomes were analyzed. Participants were divided into two groups: Group 1, composed of those who reported complete healing or improvement, and Group 2, composed of those who reported stabilization or worsening of the injury. Table 5 presents the variables that did or did not influence the evolution of the healing process.

**Table 5:** Variables analyzed to assess the relationship with the evolution of the healing process after 60 days of follow-up. Botucatu, SP, Brazil, 2022-2023

Variable	Improvement/ Healing	Stabilization/ Worsening	HR* (CI 95%)	p-value^†^
	n (%)*	n (%)*		
**Sex**			
Female	26 (49)	24 (40)	1.1 (0.8 - 1.4)	0.385
Male	27 (51)	36 (60)	-	
**Skin color**				
White	39 (74)	46 (77)	1.0 (0.7 - 1.3)	0.876
Non-white	14 (26)	14 (23)	-	
**Age**				
≥ 65 years old	26 (49)	33 (55)	1.2 (0.7 - 2.0)	0.448
< 65 years old	27 (51)	27 (45)	-	
**Hospitalization time**				
≥ 30 days	31 (59)	37 (62)	0.8 (0.5 - 1.5)	0,383
<30 days	22 (41)	23 (38)	-	
**Time until evaluation by the Wound Care Committee**				
≥ 15 days	17 (32)	23 (38)	1.0 (0.5 - 1.7)	0.881
<15 days	36 (68)	37 (62)	-	
**Number of PI^‡^**				
≥ 2	27 (51)	31 (52)	1.1 (0.8 - 1.4)	0.888
< 2	26 (49)	29 (48)	-	
**Arterial hypertension**				
Yes	37 (70)	37 (62)	1.3 (0.7 - 2.3)	0.244
No	16 (30)	23 (38)	-	
**Diabetes mellitus**				
Yes	13 (24)	20 (33)	1.2 (0.8 - 1.6)	0.202
No	40 (76)	40 (67)	-	
**Stroke**				
Yes	13 (24)	9 (15)	0.6 (0.3-1.0)	0.021
No	40 (76)	51 (85)	-	

*HR = Hazard Ratio; ^†^Log Rank Test; ^‡^PI = Pressure injury

It was found that the presence of stroke significantly influenced the healing process (HR = 0.6; 95% CI 0.3-1.0; p = 0.021). Among the patients who achieved healing within 30 days after discharge, 80% had not experienced a stroke. The absence of this comorbidity contributed to earlier healing outcomes. However, by the follow-up at D60, patients with stroke reached results similar to those without stroke, indicating that this comorbidity had a more negative impact during the early stages of the healing process.

## Discussion

This study followed patients who developed hospital-acquired PI for 60 days after discharge. In this prospective cohort, many patients experienced unfavorable outcomes, with readmission rates ranging from 10.6% to 16.8% and a mortality rate of 22.1%. These complications highlight the severity of the condition and its impact on the morbidity and mortality of patients who continue treatment for PIs. A study conducted in the United States, based on a large national database, reported that 25% of patients with hospital-acquired PI were readmitted within 30 days after discharge, and readmission rates remained high at 90 and 180 days post-discharge^23^.

At the beginning of the interviews, most patients were bedridden. However, progressive improvement in mobility was observed among those who did not experience complications and participated in the scheduled interviews throughout the 60-day follow-up. After discharge, 97.5% of patients reported adopting repositioning as a pressure-relief strategy. This measure is recognized for its effectiveness in redistributing pressure and is essential both to prevent the progression of injuries and to promote healing^24^ ^-^ ^25^.

Stroke was identified as a factor associated with unfavorable progression of the PI healing process. Patients with this condition often present impaired mobility and reduced sensitivity due to neurological compromise^26^. A study reported that the prevalence of PI among stroke patients after hospital discharge was significantly higher than during hospitalization^27^, suggesting that this group did not receive adequate care or attention for PI in the post-discharge period. Regarding the anatomical location of the injuries, the sacral region was the most affected, followed by the heels a result consistent with other studies^28^ ^-^ ^29^, which may be attributed to the predominance of the supine position during hospitalization. 

Hospital discharge is a delicate and challenging moment for patients, who often require guidance and support to manage care transitions^30^ ^-^ ^31^, particularly those with PI. Providing clear health care instructions at the time of discharge is essential^32^ in the present study, this was documented in 61 of 79 patients (77.2%). However, one investigation showed that most patients or caregivers did not have the opportunity to practice dressing change techniques during hospitalization. This gap in practical training may lead to uncertainty, increase the likelihood of errors and contribute to hospital readmissions^33^. 

In Brazil, Primary Health Care (PHC) represents the first level of care within the Unified Health System, serving as the main entry point for users into the health care network^34^ and ensuring continuity of treatment after hospital discharge. Strategies such as in-person consultations, home visits, and telemonitoring have shown a positive impact on reducing readmission rates, strengthening the bond between users and health teams, and improving adherence to therapeutic plans^35^. Conversely, late or absent follow-up after discharge may compromise the effectiveness of care transitions, increase the risk of adverse events and lead to new hospitalizations, thereby hindering the patient’s recovery process^36^. An example is the proportion of patients in the present study who remained without care, reaching 50.6% in the first week. Therefore, it is necessary to improve the counter-referral process and the integration among health services^37^, so that the responsibility for conveying information does not rest solely with the patient.

Home visits, in addition to strengthening community-based care^38^, provide individualized support for those who have difficulty traveling to health facilities, as is the case for most patients with PI. In the D7 interview, 29 patients (36.7%) reported receiving follow-up through Primary Health Care, specifically via home visits. 

Although some participants showed favorable outcomes-such as improvement (29/57; 50.8%) and complete healing (19/57; 33.3%) of PI not all achieved the same success during follow-up, mainly due to death or readmission. Living with PI imposes considerable negative impacts, including loss of independence, social isolation, dissatisfaction and the belief in failed healing, as well as several psychological effects that also extend to caregivers^38^. 

Regardless of the complexity of the injury, patients and their caregivers face significant challenges in managing PI outside the hospital setting Therefore, education and proper guidance at the time of discharge are essential and should include instructions on dressing change techniques, repositioning, use of pressure relief cushions, and other measures. Such guidance aims to equip patients and caregivers with the knowledge and skills required to effectively manage PI. Providing clear information can facilitate patient understanding and ensure that caregivers are capable of performing the necessary procedures for the continuity of home care after discharge^39^.

Failures in discharge planning, often marked by fragmented services, are associated with adverse clinical outcomes, including increased mortality, higher treatment costs, medical errors and unplanned readmissions^33^. Reducing hospital readmissions is crucial not only because of their adverse impact on patient well-being but also due to their financial implications for the health system^39^.

Most participants relied on a family member as their primary caregiver; however, six individuals in the sample (8.6%) resided in Long-Term Care Institutions for Older Adults (LTCF). Evidence indicates that the prevalence of PI among institutionalized older adults is similar to that observed in hospital settings^40^. It is well established that most older adults living in LTCF present functional dependence, particularly regarding mobility and therefore require continuous support from health professionals in general, all caregivers-whether family members or professionals working in LTCF-must align post-discharge care with the effective treatment of PI^40^.

During the study, some limitations were identified, including patient loss to follow-up, primarily related to frequent hospital readmissions and deaths. Furthermore, difficulties in conducting scheduled telephone interviews at different data collection points compromised the completeness of the dataset. The lack of data collection on participants’ educational level and housing conditions was also a limitation, as these are important social determinants that may influence the outcomes of this study. This point is particularly relevant because patients in disadvantaged socioeconomic situations tend to be more vulnerable to the development of PI^31^. Another limitation concerns the analysis of the healing process in the post-discharge period, which was based on the perceptions of patients and/or caregivers rather than on validated methods. Nevertheless, the information collected was appropriate, as it reflects the lived experience of those directly involved. Despite these limitations, the final sample size was considered adequate for the proposed analysis, without compromising validity. However, future studies with larger samples should conduct multivariate analyses of the factors that may influence the evolution of the healing process of hospital-acquired PI in the post-discharge period.

The study demonstrated both the reality of PI development in hospitalized patients and the trajectory of care and healing in the post-discharge context. This knowledge reinforces the need for strategies that enhance preventive care for at-risk patients in the hospital setting and improve quality of life after discharge. 

Implementing a structured hospital discharge plan for patients who develop PI is recommended, with the use of clear and accessible language, illustrations, and detailed instructions on dressing changes and pressure relief measures. Strengthening communication mechanisms among health teams is also essential to ensure continuity and alignment of care, regardless of the level of care to which the patient is transferred^19^ ^,^ ^33^.

## Conclusion

During the 60-day post-discharge follow-up of patients with hospital-acquired PI, approximately 22% died. Readmission rates ranged from 10.6% to 16.8% during this period. A substantial proportion of patients did not receive adequate support from health services for PI care. Conversely, among those followed up to D60, most showed improvement or complete healing of their injuries. Patients with stroke, however, experienced a slower progression of the healing process.

These findings underscore the urgent need to strengthen interventions and post-discharge follow-up to optimize recovery and healing, with the active involvement of patients, families, and caregivers. Identifying high-risk patients, such as those with stroke, and reinforcing preventive strategies are essential to improve both the quality of care and patient outcomes. Finally, health education initiatives, the development of innovative solutions, and advances in care delivery can enhance the effectiveness of PI management and healing in the home setting.

## Data Availability

All data generated or analysed during this study are included in this published article.

## References

[1] Brienza D, Gefen A, Clark M, Black J (2022). The vision and scope of the prophylactic dressing standard initiative of the European Pressure Ulcer Advisory Panel and National Pressure Injury Advisory Panel. Int Wound J.

[2] Siotos C, Bonett AM, Damoulakis G, Becerra AZ, Kokosis G, Hood K (2022). Burden of pressure injuries: findings from the Global Burden of Disease Study.

[3] Li Z, Lin F, Thalib L, Chaboyer W (2020). Global prevalence and incidence of pressure injuries in hospitalised adult patients: a systematic review and meta-analysis. Int J Nurs Stud.

[4] Agência Nacional de Vigilância Sanitária (BR) (2023). Relatórios de notificação dos estados - eventos adversos: 2023.

[5] Cox J, Edsberg LE, Koloms K, VanGilder CA (2022). Pressure injuries in critical care patients in US hospitals: results of the International Pressure Ulcer Prevalence Survey. J Wound Ostomy Continence Nurs.

[6] Isfahani P, Alirezaei S, Samani S, Bolagh F, Heydari A, Sarani M (2024). Prevalence of hospital-acquired pressure injuries in intensive care units of the Eastern Mediterranean region: a systematic review and meta-analysis. Patient Saf Surg.

[7] Rapetti R, Pansera A, Visca S, Martolini M, Antoniotti S, Bertoncini F (2023). Pressure ulcers in hospital patients: incidence and risk factors. J Wound Care.

[8] Vieira S, Mostardinha A, Alves P. (2024). Unveiling the burden: a six-year retrospective analysis of pressure ulcer epidemiology in an ICU. Nurs Rep.

[9] Chen B, Yang Y, Cai F, Zhu C, Lin S, Huang P (2023). Nutritional status as a predictor of the incidence of pressure injury in adults: a systematic review and meta-analysis. J Tissue Viability.

[10] Roussou E, Fasoi G, Stavropoulou A, Kelesi M, Vasilopoulos G, Gerogianni G (2023). Quality of life of patients with pressure ulcers: a systematic review. Med Pharm Rep.

[11] Thomas DC, Chui PL, Yahya A, Yap JW (2022). Systematic review of patient education for pressure injury: evidence to guide practice. Worldviews Evid Based Nurs.

[12] Ibemere SO, You H, McReynolds V, Huang M, Anaya B, Gonzalez-Guarda RM (2024). Challenges in the transition from acute hospital care to home for Spanish-speaking Latino patients with TBI and families: perspectives of healthcare providers and interpreters. J Racial Ethn Health Disparities.

[13] Collier M, Jones S, Glendewar G (2023). Pressure ulcer prevention, patient positioning and protective equipment. Br J Nurs.

[14] National Pressure Injury Advisory Panel, European Pressure Ulcer Advisory Panel, Pan Pacific Pressure Injury Alliance (2019). Prevention and treatment of pressure ulcers/injuries: clinical practice guideline.

[15] Qian L, Yan S, Ting ST, Han ZM, Qi T (2024). Complications and psychological impact of pressure ulcers on patients and caregivers. Int Wound J.

[16] Sen H, Kilic M (2024). Determination of the family caregivers’ level of knowledge on pressure injury prevention. J Tissue Viability..

[17] von Elm E, Altman DG, Egger M, Pocock SJ, Gøtzsche PC, Vandenbroucke JP (2007). The strengthening the reporting of observational studies in epidemiology (STROBE) statement: guidelines for reporting observational studies. BMJ.

[18] Bergstrom N, Smout R, Horn S, Spector W, Hartz A, Limcangco MR (2008). Stage 2 pressure ulcer healing in nursing homes. J Am Geriatr Soc.

[19] Bergstrom N, Braden BJ, Laguzza A, Holman V (1987). The Braden scale for predicting pressure sore risk. Nurs Res.

[20] Miot HA (2017). Assessing normality of data in clinical and experimental trials. J Vasc Bras..

[21] Dudley WN, Wickham R, Coombs N (2016). An introduction to survival statistics: Kaplan-Meier analysis. J Adv Pract Oncol.

[22] Miot HA (2017). Survival analysis in clinical and experimental studies. J Vasc Bras.

[23] Dreyfus J, Gayle J, Trueman P, Delhougne G, Siddiqui A (2018). Assessment of risk factors associated with hospital-acquired pressure injuries and impact on health care utilization and cost outcomes in US hospitals. Am J Med Qual.

[24] Sardo PMG, Teixeira JPF, Machado AMSF, Oliveira BF, Alves IM (2023). A systematic review of prevalence and incidence of pressure ulcers/injuries in hospital emergency services. J Tissue Viability.

[25] Zhou L, Hu Y, Ma D, Ren B, Cui J, Zhou Q (2024). Best evidence summary for the prevention of pressure injuries in orthopaedic patients. J Clin Nurs.

[26] Soares LCB, Silva DO, Cunha JXP, Pires PS, Cardoso LGV (2022). Pressure injury development and care complexity in patients at an emergency service. Cogitare Enferm.

[27] Vanaki Z, Mohammadi E, Hosseinzadeh K, Ahadinezhad B, Rafiei H (2023). Prevalence of pressure injury among stroke patients in and out of healthcare settings: a systematic review and meta-analysis. Home Healthc Now.

[28] Castro DLV, Santos VLCG, Chicone G, Rocha MNB, Nogueira PC, Serpa LF (2022). Prevalence of pressure injury and associated factors in hospitalized adult patients with cancer. Wounds.

[29] Al-Mamari F, Al-Rawajfah O, Al Sabei S, Al-Wahaibi K (2024). Hospital-acquired pressure ulcers among adult ICU patients in tertiary hospitals in Oman: a one-year prevalence study. J Wound Care.

[30] Aloweni F, Gunasegaran N, Lim SH, Leow BWX, Agus N, Goh IHQ (2024). Socio-economic and environmental factors associated with community-acquired pressure injuries: a mixed method study. J Tissue Viability.

[31] Hindsbak N, Morsø L, Hvidtjørn D, Walløe S (2024). Identification of interventions to improve patient experienced quality of care in transitions between healthcare settings: a scoping review.. BMC Health Serv Res.

[32] Trivedi SP, Corderman S, Berlinberg E, Schoenthaler A, Horwitz LI (2023). Assessment of patient education delivered at time of hospital discharge. JAMA Intern Med.

[33] Ramos FT, Meira JRR, Colenci R, Alencar RA (2021). Association between the orientation received during hospitalization and the occurrence of wound healing. Rev Bras Enferm.

[34] Macedo TR, Calvo MCM, Possoli L, Natal S (2022). Patient safety in primary health care: a look at the literature. Rev APS.

[35] Santos MM, Peradotto BC, Micheletti VCD, Treviso P (2022). Transition from tertiary care to primary care: integrative literature review. Nursing.

[36] Rocha MNT, Calheiros DS, Wyszomirska RMF (2022). The referral and counter-referral system in mental health from the perspective of the physician working in primary care. Res Soc Dev.

[37] Eltaybani S, Kawase K, Kato R, Inagaki A, Li CC, Shinohara M (2023). Effectiveness of home visit nursing on improving mortality, hospitalization, institutionalization, satisfaction, and quality of life among older people: umbrella review. Geriatr Nurs.

[38] Tobiano G, Walker RM, Chaboyer W, Carlini J, Webber L, Latimer S (2023). Patient experiences of, and preferences for, surgical wound care education. Int Wound J.

[39] Dhaliwal JS, Dang AK (2025). Reducing hospital readmissions. StatPearls.

[40] Sugathapala RDUP, Latimer S, Balasuriya A, Chaboyer W, Thalib L, Gillespie BM (2023). Prevalence and incidence of pressure injuries among older people living in nursing homes: a systematic review and meta-analysis. Int J Nurs Stud.

