# Supplementary material for: Post-discharge evaluation of patients with hospital-acquired pressure injuries: prospective cohort study
Source: Rev Lat Am Enfermagem. 2026 Jun 15;34:e4768. doi: 10.1590/1518-8345.7866.4768 (PMC13277348; doi:10.1590/1518-8345.7866.4768)
Supplement: Translation

# Translation: Avaliação pós-alta de pacientes com lesão por pressão hospitalar: estudo de coorte prospectivo^*^

**DOI:** 10.1590/1518-8345.7866.4769

**Authors:** Michelle Venâncio Hong, Bruna Cristina Velozo, Isabela Novello, Hélio Amante Miot, Meire Cristina Novelli e Castro, Luciana Patrícia Fernandes Abbade

**Affiliations:** 1 Universidade Estadual Paulista Júlio de Mesquita Filho, Faculdade de Medicina de Botucatu, Departamento de Enfermagem, Botucatu, SP, Brasil.; 2 Universidade Estadual Paulista Júlio de Mesquita Filho, Faculdade de Medicina de Botucatu, Departamento de Dermatologia, Botucatu, SP, Brasil.

**Keywords:** Lesão por Pressão, Alta do Paciente, Serviços de Assistência Domiciliar, Assistência Domiciliar, Continuidade da Assistência ao Paciente, Cicatrização.

## Abstract

**Objetivo:**

avaliar a evolução cicatricial das lesões por pressão desenvolvidas no ambiente hospitalar de pacientes adultos no período pós-alta, assim como as intercorrências e cuidados direcionados a esses pacientes

**Método:**

estudo de coorte prospectivo com pacientes que desenvolveram lesão por pressão durante internação e receberam alta hospitalar. As informações foram coletadas em 7, 15, 30 e 60 dias após a alta hospitalar.

**Resultado:**

a amostra incluiu 113 pacientes, sendo 63 homens (55,8%), com média de idade 64,6 anos (± 15,1). Ocorreram 246 lesões, predominantemente na região sacral (91; 37,0%) e de estágio 2 (117; 47,6%). As taxas de reinternação variaram entre 10,6% e 16,8% ao longo de 60 dias e ocorreram 25 (22,1%) óbitos. Na última entrevista, 57 pacientes participaram, dos quais 29 (50,9%) permaneciam acamados ou com mobilidade muito limitada e 48 (84,2%) tiveram melhora ou cicatrização da lesão. O acidente vascular encefálico ocorreu em 22 (19,5%) pacientes e demonstrou ser um fator desfavorável ao processo de cicatrização (HR = 0,6; p=0,021).

**Conclusão:**

durante 60 dias após a alta, os pacientes tiveram desfechos desfavoráveis, como óbito, readmissão hospitalar e não cicatrização das lesões por pressão. Pacientes com acidente vascular encefálico tiveram evolução mais lenta do processo cicatricial.

## Destaques

**(1)** Após a alta hospitalar 22% dos pacientes evoluíram para óbito. **(2)** As taxas de reinternação variaram entre 10,6% e 16,8%. **(3)** A presença da comorbidade acidente vascular encefálico afetou desfavoravelmente o processo de cicatrização. **(4)** No 60º dia pós-alta, 50,9% das lesões por pressão melhoraram e 33,3% cicatrizaram totalmente. **(5)** Após a alta, 50,6% dos pacientes não tiveram assistência na primeira semana.

## Introdução

A lesão por pressão (LP) caracteriza-se pela ocorrência de danos à pele e/ou tecidos subjacentes mediante a aplicação de força externa à pele em áreas de proeminência óssea ou por meio do uso de dispositivos médicos ou outros objetos. Frequente em hospitais, a LP é considerada um evento adverso, sendo um indicador da qualidade da assistência prestada nos serviços de saúde^1^.

Apesar dos avanços nas estratégias de prevenção, a incidência de LP tem se mantido relativamente estável nos últimos anos^2^. Estima-se globalmente a prevalência de 12,8% e a incidência de 8,5% de LPs adquiridas por adultos hospitalizados^3^. No Brasil, mais de 60 mil casos foram notificados em 2023^4^. Há variações significativas na incidência entre os diversos setores hospitalares, sendo que as Unidades de Terapia Intensiva (UTI) representam as maiores taxas de prevalência (14,3%)^5^ ^-^ ^6^.

A LP pode ocorrer devido aos fatores inerentes ao paciente, tais como idade avançada, fragilidade da pele^7^, mobilidade reduzida, cisalhamento, hospitalizações prolongadas^8^ e desnutrição, a qual constitui um fator de risco relevante^9^. Além de representar um desafio constante nos cuidados de saúde, a LP é uma das questões clínicas mais difíceis de ser manejadas. Ela afeta negativamente os pacientes do ponto de vista emocional, mental, físico e social, resultando em uma redução geral da qualidade de vida^10^. 

A LP frequentemente aumenta a dependência dos sistemas de saúde e das redes de apoio^10^, repercutindo em maior demanda de cuidados tanto na internação quanto na transição de cuidados para alta hospitalar. Na alta, há uma vulnerabilidade relacionada à segurança do paciente e de sua família, por causa da possível inconsistência de informações importantes para cicatrização, como cuidados tópicos, medidas de alívio da pressão e avaliação frequente da pele^11^.

As mudanças no ambiente decorrentes da hospitalização impactam não apenas o paciente, mas também seus familiares ou cuidadores. O retorno ao lar com uma LP não só implica modificações na rotina diária, mas também adiciona outras responsabilidades no ambiente doméstico^12^. São recomendados objetos de apoio, mobília adequada^13^, espaço físico organizado para higiene e mobilidade segura, além de cuidados com a prevenção de quedas^14^. Essas modificações, aliadas ao apoio dos cuidadores, garantem um cuidado eficaz e seguro no domicílio. No entanto, elas frequentemente não são discutidas durante a internação e alta hospitalar, o que pode comprometer a assistência aos pacientes com LP e dificultar a cicatrização^15^.

Sabe-se que é necessária a orientação adequada aos familiares e cuidadores quanto ao tratamento e prevenção de LP. Nesse sentido, a padronização de *guidelines* pós-alta permite amenizar carências de conhecimento sobre o tema^16^. Além do que, os cuidados dos pacientes com LP são extremamente complexos, principalmente para pessoas leigas, levando aos desfechos desfavoráveis como readmissões hospitalares, complicações e declínio na qualidade de vida^15^. 

Mesmo diante da relevância do tema, faltam estudos que abordem os desfechos pós-alta de pacientes com LP adquiridas nos serviços de saúde e o modo pelo qual os familiares ou cuidadores informais acompanham essas lesões em domicílio. Dessa maneira, é relevante realizar pesquisas sobre estes desfechos em pacientes que deixam o ambiente hospitalar, bem como investigar as suas características evolutivas no ambiente extra-hospitalar. 

Portanto, o objetivo deste estudo foi avaliar a evolução cicatricial das LP desenvolvidas no ambiente hospitalar de pacientes adultos no período pós-alta, assim como as intercorrências e cuidados direcionados a esses pacientes.

## Método

### Tipo do estudo

Trata-se de um estudo de coorte prospectivo, relatado conforme as diretrizes *Strengthening the Reporting of Observational studies in Epidemiology* (STROBE)^17^.

### Participantes e local do estudo

O estudo incluiu pacientes adultos que desenvolveram LP durante a internação em enfermarias ou UTI de dois hospitais públicos de ensino localizados na cidade de Botucatu, no interior de São Paulo, Brasil. Ambos os hospitais pertencem à mesma administração: um é de nível secundário e o outro é de nível terciário.

As instituições contam com a mesma Comissão de Curativos, composta por enfermeiros especializados que atendem os dois hospitais na assistência aos pacientes com LP ou outras lesões que demandam cuidados específicos. Os pacientes com LP, acompanhados durante a internação e que recebem alta, são orientados pela Comissão de Curativos e por enfermeiros assistenciais para a alta segura. É entregue uma carta de contrarreferência para acompanhamento na Atenção Primária, compreendendo orientações e recomendações dos curativos realizados. 

No estudo, foram considerados os pacientes avaliados pela Comissão de Curativos e que receberam alta hospitalar entre janeiro de 2022 e janeiro de 2023. 

### Critérios de elegibilidade

Os critérios de inclusão na pesquisa foram: pacientes que desenvolveram LP hospitalar de ambos os sexos, com idade superior a 18 anos, que receberam avaliação da Comissão de Curativos das instituições ao longo do período de internação hospitalar e que tiveram alta hospitalar com LP.

Foram excluídos os que já apresentavam LP quando foram admitidos no hospital, mesmo que tenham desenvolvido novas lesões durante a internação e os pacientes com LP que foram transferidos para instituições não incluídas no estudo.

### Desfechos

O desfecho principal foi a avaliação dos fatores associados à evolução do processo cicatricial no período de 60 dias após a alta hospitalar. Segundo a percepção dos cuidadores, os participantes foram divididos em dois grupos: evolução favorável do processo cicatricial (cicatrização total ou melhora) e evolução desfavorável do processo cicatricial (estável ou piora). Foram atribuídos os seguintes critérios nas entrevistas realizadas com pacientes, familiares ou cuidadores: (i) Sinais de melhora: presença de ferida com tecido avermelhado (tecido de granulação), diminuição do exsudato e redução de área e (ii) Sinais de piora: aumento de necrose, odor, dor, exsudato e área inalterada ou aumentada.

Os desfechos secundários foram: (i) Descrever as características basais sociodemográficas, clínicas e terapêuticas dos pacientes que desenvolveram LP em ambiente hospitalar; (ii) Relatar as intercorrências (óbito e reinternação) e as variáveis relacionadas aos cuidados dos pacientes no período de acompanhamento pós-alta, as quais foram obtidas nas entrevistas.

### Instrumentos de coleta de dados 

Para a busca ativa dos pacientes, foi feita uma pesquisa entre os relatórios da Comissão de Curativos no sistema do hospital. Neles, verificava-se se a LP era domiciliar ou hospitalar, selecionando todos os pacientes que desenvolveram LP intra-hospitalar. 

Para avaliar a conformidade dos critérios de elegibilidade, era acessado o prontuário eletrônico em busca de informações do paciente. Após averiguação e consentimento de participação por meio da obtenção do Termo de Consentimento Livre e Esclarecido (TCLE), iniciavam-se as entrevistas com os pacientes, familiares ou cuidadores via telefone. Nas entrevistas, eram obtidas informações sobre orientações de alta e adesão às orientações, curativo utilizado, acesso aos serviços de saúde, condições clínicas gerais e processo evolutivo das LP.

### Coleta de dados e variáveis do estudo

A coleta das informações referiu-se ao período de internação (variáveis coletadas por meio do prontuário eletrônico) e aos dias 7 (D7), 15 (D15), 30 (D30) e 60 (D60) após a alta hospitalar (variáveis coletadas por meio de entrevistas). O período de acompanhamento máximo de 60 dias foi estabelecido considerando a viabilidade da coleta das informações necessárias nesse intervalo para avaliar os cuidados extra-hospitalares e evolução do processo cicatricial. Essa definição baseou-se em um estudo de coorte retrospectivo que identificou o tempo médio de 46 dias para cicatrização de LP^18^. 

Se o participante ou cuidador não atendesse o telefone em três tentativas de ligação, considerava-se que a entrevista do dia não tinha sido realizada. No entanto, o participante permanecia no estudo e participava das entrevistas subsequentes até a conclusão. Por ser um estudo observacional, não havia interferência na rotina de assistência a esses pacientes nem nas orientações de alta.

Para coletar os dados, foi utilizado um formulário online (Google Forms^®^) criado pelos pesquisadores e validado após um teste-piloto com dez pacientes. O formulário final incluiu variáveis dependentes e independentes do estudo, como:

- Dados sociodemográficos: sexo, idade, cor/raça declarada (branca, negra, parda, amarela e/ou outra) e renda familiar;
- Variáveis clínicas: comorbidades relevantes, tempo de internação (em dias), origem do paciente antes da hospitalização, motivo da internação, complicações durante a internação e tempo de internação (em dias) até acionar a Comissão de Curativos para avaliação da LP;
- Quantidade de LP desenvolvidas por paciente, localização anatômica e classificação;
- Abordagem terapêutica da LP no ambiente hospitalar.

- Entrevista pós-alta: intercorrências (óbito e reinternação); local de instalação do paciente pós-alta (residência própria, residência de familiar e/ou amigos e Instituições de Longa Permanência para Idosos - ILPI); cuidadores (familiar, amigo, técnico/auxiliar de enfermagem ou cuidador capacitado sem formação); se houve orientação após a alta hospitalar; adesão às orientações pós-alta (questionado se aderiu de forma completa, incompleta ou não aderiu); locais de assistência à saúde após alta; tipo e frequência da assistência; mobilidade que o paciente apresentava no momento da entrevista segundo a dimensão “mobilidade” da Escala de Braden^(19)^; medidas adotadas para tratamento pós-alta das LPs; curativo utilizado no momento da entrevista e evolução pós-alta do processo cicatricial da LP, obtido segundo a visão do paciente/cuidador (cicatrização total, melhora, estabilização ou piora).

### Definição da amostra

Segundo dados anteriores da Comissão de Curativos dos hospitais incluídos no estudo, ocorreram 269 LP de origem hospitalar entre outubro de 2019 e outubro de 2020. O tamanho amostral determinado para o estudo foi por conveniência, considerando a coleta de dados durante 12 meses para inclusão de participantes no estudo.

### Análise de dados

Foi realizada estatística descritiva dos dados usando frequência e porcentagem para as variáveis categóricas e utilizando média, desvio-padrão, mediana, mínimo e máximo para as variáveis quantitativas. A normalidade das distribuições das variáveis quantitativas (contínuas) foi avaliada pelo teste de Shapiro-Wilk e os resultados dessas variáveis foram apresentados como médias (± desvio-padrão) quando houve distribuição normal e como mediana (p25 - p75) quando a distribuição não foi normal^20^.

Foram separados dois grupos para análise: o grupo com evolução favorável (cicatrização total e melhora da LP) e o grupo com evolução desfavorável (LP estável ou com piora). A análise da curva de sobrevivência (Kaplan-Meier)^21^ com uso do teste de *Log Rank* (Mantel-Cox) foi realizada para identificar os fatores que influenciam a evolução favorável (melhora/cicatrização). Nos casos com diferença na sobrevida, a dimensão do efeito foi estimada pelo *Hazard Ratio* (HR) e seu intervalo de confiança de 95% (IC 95%)^22^. Foram censurados os casos que não atingiram o desfecho de evolução favorável por terem saído do estudo em razão de alguma perda de seguimento (óbito, reinternação ou não realização da entrevista).

As variáveis analisadas foram escolhidas de acordo com suas possíveis influências no processo cicatricial e dicotomizadas segundo suas frequências médias, tais como sexo, idade acima de 65 anos, cor/raça branca versus não brancos, período de internação de 30 dias, tempo mínimo de 15 dias para avaliação pela Comissão de Curativos, quantidade de até duas LP e comorbidades (hipertensão arterial [HA], diabetes *mellitus* [DM] e acidente vascular encefálico [AVE]).

Todas as análises estatísticas foram efetuadas nos programas SPSS 23.0 (IBM). Foi considerado o intervalo de confiança (IC) de 95% e a significância de 5% (p < 0,05).

### Aspectos éticos

Este estudo foi aprovado pelo Comitê de Ética em Pesquisa e reconhecido pela Comissão Nacional de Ética em Pesquisa sob Certificado de Apresentação de Apreciação Ética nº 53331521.6.0000.5411, atendendo às normas das Resoluções n° 466/2012 e n° 510/2016 do Conselho Nacional de Saúde. Após o esclarecimento e orientações sobre a pesquisa, a participação foi condicionada ao prévio consentimento do paciente e/ou responsável mais próximo. Quando o paciente não se encontrava nas instituições em decorrência da alta hospitalar, obtinha-se o TCLE remotamente. 

## Resultados

No período do estudo, o relatório do sistema hospitalar registrou 962 avaliações com o diagnóstico de “Lesão por Pressão Hospitalar”, feitas pela Comissão de Curativos. Após remover as duplicatas, ou seja, os pacientes readmitidos em momentos diferentes, totalizaram-se 461 pacientes. Foram excluídos aqueles que não atendiam aos critérios de elegibilidade, ou seja, os que evoluíram ao óbito hospitalar (n = 248), menores de 18 anos (n = 27), transferidos para outra unidade hospitalar (n = 9), apresentavam LP no momento da admissão hospitalar ou outro tipo de lesão (n = 35) e aqueles aos quais não se solicitou interconsulta da Comissão de Curativos (n = 9). Portanto, 133 pacientes eram elegíveis, porém 113 consentiram em participar e integraram o estudo.

### Características sociodemográficas, clínicas e terapêuticas dos pacientes que desenvolveram LP em ambiente hospitalar

Na Tabela 1, são apresentados os dados sociodemográficos e clínicos dos 113 participantes. A maioria, do sexo masculino (63; 55,8%), média de idade de 64,6 (± 15,1) anos, cor/raça branca autodeclarada (85; 75,2%) e renda familiar de um a três salários mínimos (49; 43,3%). A principal comorbidade foi a HA (74; 65,5%). O tempo de internação variou de 7 a 148 dias, com mediana de 40,0 (22,0 - 57,0). Antes da hospitalização, 63 (55,8%) encontravam-se no domicílio próprio e as principais razões da internação relacionavam-se às lesões por causa externa (25; 22,1%) e doenças do sistema nervoso (24; 21,2%). Infecções (52; 46,0%) foram as complicações mais observadas. 

**Tabela 1:** Dados sociodemográficos e clínicos dos participantes com lesão por pressão hospitalar (n = 113). Botucatu, SP, Brasil, 2022-2023

**Variáveis**	**n (%)**
**Sexo**		
Masculino	63 (55,8)
Feminino	50 (44,2)
**Idade em anos**		
Mínimo e máximo	22 a 95
Média (± desvio-padrão)	64,6 (15,1)
**Cor/raça**		
Branca	85 (72,2)
Negra	17 (15,0)
Parda	11 (9,7)
**Renda familiar**		
Menos que um SM*	9 (7,9)
Um a três SM*	49 (43,3)
Quatro a dez SM*	21 (18,5)
Não informado	34 (30)
**Comorbidades^†^**		
Hipertensão arterial	74 (65,5)
Diabetes mellitus	34 (30,1)
Acidente vascular encefálico	22 (19,5)
**Período de internação em dias**		
Mínimo e máximo	7 a 148
Mediana (p25 - p75)	40,0 (22,0 - 57,0)
**Procedência antes da hospitalização**		
Residência própria	74 (65,5)
Transferido de outra hospitalização	32 (28,3)
Instituições de Longa Permanência para Idosos	5 (4,4)
Sem resposta	2 (1,8)
**Motivo da internação^†^**		
Lesões por causas externas^‡^	25 (22,1)
Doença do sistema nervoso	24 (21,2)
Doença do aparelho respiratório	21 (18,6)
Doença do aparelho circulatório	19 (16,8)
Neoplasias	11 (9,7)
Doenças endócrinas, metabólicas e nutricionais	10 (8,8)
Doença do aparelho geniturinário	10 (8,8)
Doença do aparelho digestivo	8 (7,1)
Doença autoimunes	2 (1,8)
**Complicações durante a internação^†^**		
Infecções	52 (46,0)
Complicações respiratórias	43 (38,1)
Parada cardiorrespiratória	10 (8,8)
Complicações cardiovasculares	10 (8,8)
Teste de COVID-19 positivo	10 (8,8)
Trombose venosa profunda	3 (2,7)
Acidente vascular encefálico	2 (1,8)
Infarto agudo do miocárdio	1 (0,9)
Sem complicações	10 (8,8)

*SM = Salário-mínimo vigente em 2022 (R$ 1.212,00), Brasil; ^†^Mais de um resultado para cada paciente; ^‡^Lesões por causas externas: fraturas, quedas, agressões e acidentes envolvendo transporte

A Comissão de Curativos foi acionada após mediana de 13,0 (6,0 - 29,0) dias de internação. Foram identificadas 246 LP em 113 pacientes, a maioria dos quais com apenas uma lesão (58; 51,3%). A região sacral (91; 37,0%) e o calcâneo (49; 19,9%) foram as localizações anatômicas mais acometidas e a classificação da lesão mais identificada foi a de estágio 2 (117; 47,6%) (Tabela 2).

A Tabela 3 mostra as abordagens terapêuticas e medidas de alívio de pressão adotadas. Identifica-se que o tratamento tópico mais prescrito foi o polihexametileno de biguanida em líquido e/ou gel (109; 44,3%). A medida preventiva mais recomendada foi o reposicionamento no leito de duas em duas horas (173; 70,3%). Destaca-se que o colchão pneumático foi indicado para apenas 38 participantes (15,4%).

**Tabela 2:** Características das 246 lesões por pressão (LPs*) hospitalares (n = 113). Botucatu, SP, Brasil, 2022-2023

**Variáveis**	**n (%)**
**Relacionadas aos pacientes**	**n = 113**
**Tempo de internação (em dias) até acionar a Comissão de Curativos**		
Mínimo e máximo	1 a 105
Mediana (p25 - p75)	13,0 (6,0 - 29,0)
**Quantidade de lesões por pressão desenvolvidas por paciente**		
Uma	58 (51,3)
Três	19 (16,8)
Mais de três	19 (16,8)
Duas	17 (15,0)
**Relacionadas às lesões por pressão**	**n = 246**
**Localização anatômica**	
Sacral	91 (37,0)
Calcâneo	49 (19,9)
Isquiática	21 *(*8,5)
Tornozelo	17 (6,9)
Pavilhão auricular	10 (4,1)
Trocânter	10 (4,1)
Pés ou mãos	8 (3,3)
Occipital	8 (3,3)
Outras regiões anatômicas*	32 (14,1)
**Classificação das lesões por pressão**		
Estágio 2	117 (47,6)
Estágio 1	60 (24,4)
Lesão não classificável	31 (12,6)
Estágio 3	12 (4,9)
Lesão tissular profunda	11 (4,5)
Lesão por dispositivo médico	11 (4,5)
Estágio 4	4 (1,6)

*Outras regiões anatômicas: maléolo lateral ou medial, coluna, escapular, tibial, nasal, joelho, lábios e cotovelo

**Tabela 3:** Abordagem terapêutica tópica e orientação de alívio de pressão para os 113 pacientes com 246 lesões por pressão hospitalares. Botucatu, SP, Brasil, 2022-2023

**Variáveis**	**n (%)**
Tratamento tópico no ambiente hospitalar^*^		
Polihexametileno de biguanida (PHMB) líquido ou gel	109 (44,3)
Hidrogel	65 (26,4)
Protetores cutâneos^†^	89 (36,2)
Papaína	21 (8,5)
Alginato de cálcio em placa	12 (4,9)
Espuma de poliuretano/hidropolímero em placa	9 (3,7)
Colagenase	8 (3,3)
Ácidos graxos essenciais	6 (2,4)
Hidrofibra com prata	6 (2,4)
Hidrocoloide	3 (1,2)
Sulfadiazina de prata	2 (0,8)
Curativo de pressão negativa	1 (0,4)
Orientações da Comissão de Curativos*		
Reposicionamento no leito de duas em duas horas	77 (68,1)
Almofada/coxins	30 (26,5)
Hidratação cutânea e/ou realizar medidas de prevenção	29 (25,6)
Colchão piramidal	27 (23,9)
Realizar troca diária de curativo	23 (20,3)
Alívio de calcâneos	17 (15,0)
Protetores cutâneos^†^	17 (35,4)
Colchão pneumático	14 (12,4)
Sem orientações	2 (1,7)

*Cada paciente poderia ter mais de um tratamento ou orientação prescrita durante a internação; ^†^Protetores cutâneos (creme barreira, filme spray e/ou filme poliuretano)

### Resultados do período de acompanhamento pós-alta

As variáveis das entrevistas durante o período de 60 dias pós-alta hospitalar estão apresentadas na Tabela 4. Foram constatados 25/113 (22,1%) óbitos durante esse intervalo. O número de reinternação variou em cada entrevista mas, na primeira semana após a alta, 19 (16,8%) pacientes retornaram ao hospital e, no D60, 12 (10,6%). No decorrer das entrevistas, o número de pacientes/cuidadores entrevistados diminuiu devido às intercorrências (óbito, reinternação ou não realização da entrevista por não responder às ligações telefônicas). Inicialmente, foram entrevistados 79 (69,9%), mas, ao final, havia 57 (50,4%) participantes da amostra total.

**Tabela 4:** Variáveis relacionadas aos 113 pacientes com lesões por pressão hospitalares no período de 60 dias após a alta hospitalar. Botucatu, SP, Brasil, 2022-2023

**Variáveis**	D7* n (%)	D15* n (%)	D30* n (%)	D60* n (%)
**Não entrevistados - Motivos**	34 (30)	38 (33,6)	36 (31,8)	36 (35,4)
Óbito^†^	5 (4,4)	10 (8,8)	15 (13,3)	25 (22,1)
Reinternação	19 (16,8)	17 (15,0)	19 (16,8)	12 (10,6)
Não respondeu ao contato telefônico	10 (8,8)	16 (14,2)	12 (10,6)	19 (16,8)
**Entrevistados**	79 (69,9)	70 (61,9)	67 (59,3)	57 (50,4)
**Local de instalação**				
Residência própria	46 (58,2)	41 (58,6)	43 (64,2)	36 (63,2)
Residência familiar e/ou de amigos	28 (35,4)	23 (32,9)	19 (28,4)	18 (31,6)
Instituições de Longa Permanência para Idosos	5 (6,3)	6 (8,6)	5 (7,5)	3 (5,3)
**Cuidadores^‡^**				
Familiar	74 (75,5)	64 (68,8)	61 (70,9)	51 (89,5)
Cuidador sem formação	13 (13,3)	16 (17,2)	16 (18,6)	8 (14,0)
Técnico/ou auxiliar de enfermagem	9 (9,2)	11 (11,8)	8 (9,3)	4 (7,0)
Amigo	2 (2,0)	2 (2,2)	1 (1,2)	1 (1,8)
**Houve orientação sobre lesões por pressão**				
Sim	61 (77,2)	62 (88,6)	55 (82,1)	49 (86,0)
Não	18 (22,8)	8 (11,4)	12 (17,9)	8 (14,0)
**Adesão à orientação de lesões por pressão**				
Sim, completamente	57 (72,2)	59 (84,3)	54 (80,6)	48 (84,2)
Sim, parcialmente	4 (5,1)	4 (5,7)	1 (1,5)	0 (0,0)
Não	18 (22,8)	7 (10,0)	12 (17,9)	9 (15,8)
**Assistência de cuidados^‡^**				
Sem assistência	40 (50,6)	23 (32,9)	23 (34,3)	20 (35,1)
Atenção Primária à Saúde	29 (36,7)	28 (40,0)	20 (29,9)	19 (33,3)
Ambulatório Especializado e/ou Clínica Privada	7 (8,9)	23 (32,9)	25 (37,3)	19 (33,3)
Pronto-socorro	4 (5,1)	NA	1 (1,5)	1 (1,8)
**Tipo de assistência^‡^**				
Sem assistência	40 (50,6)	23 (32,9)	23 (34,3)	20 (35,1)
Visita domiciliar	30 (38,0)	29 (41,4)	24 (35,8)	20 (35,1)
Atendimento no próprio local de saúde	9 (11,4)	19 (27,1)	22 (32,8)	18 (31,6)
**Frequência da assistência**				
Sem assistência	40 (50,6)	23 (32,9)	23 (34,3)	20 (35,1)
Semanal	32 (40,5)	26 (37,1)	21 (31,3)	16 (28,1)
Quinzenal	-	21 (30,0)	15 (22,4)	5 (8,8)
Mensal	-	-	10 (14,9)	16 (28,1)
**Mobilidade**				
Acamado	40 (50,6)	26 (37,1)	23 (34,3)	16 (28,1)
Muito limitado	25 (31,6)	28 (40,0)	25 (37,3)	13 (22,8)
Discreta limitação	9 (11,4)	13 (18,6)	11 (16,4)	18 (31,6)
Sem limitação	5 (6,3)	3 (4,3)	8 (11,9)	10 (17,5)
**Medidas de alívio de pressão^‡^**				
Reposicionamento	77 (97,5)	68 (97,1)	61 (91,0)	46 (80,7)
Coxins	31 (39,2)	30 (42,9)	29 (43,3)	24 (42,1)
Colchões do tipo piramidal e pneumático	17 (21,5)	23 (32,9)	21 (31,3)	10 (17,5)
**Curativo utilizado^‡^**				
Creme barreira	37 (46,8)	28 (40,0)	28 (41,8)	18 (31,6)
Hidrogéis/papaínas	37 (46,8)	34 (48,6)	41 (58,6)	20 (35,1)
Ácido graxo essencial	26 (32,9)	24 (34,3)	19 (28,4)	14 (24,6)
Sem utilização de produto	3 (3,8)	4 (5,7)	8 (11,9)	13 (22,8)
Outros^§^	12 (15,2)	14 (20,0)	7 (10,4)	14 (24,6)
**Evolução do processo cicatricial**				
Melhora	53 (67,1)	54 (77,1)	49 (73,1)	29 (50,9)
Estabilização	20 (25,3)	8 (11,4)	3 (4,5)	6 (10,5)
Cicatrização total	5 (6,3)	6 (8,6)	11 (16,4)	19 (33,3)
Piora	1 (1,3)	2 (2,9)	4 (6,0)	3 (5,3)

*D7, D15, D30 e D60: Referem-se aos dias de acompanhamento após a alta hospitalar, respectivamente 7º, 15º, 30º e 60º dia; ^†^Óbito: a partir do D15, o total de óbitos passou a representar a somatória acumulada dos eventos ocorridos nas visitas anteriores; ^‡^Mais de um resultado para cada paciente; ^§^Outros Curativos: filme spray, colagenase, placa de alginato de cálcio e sódio e polihexametileno de biguanida (PHMB) em líquido ou gel

A própria residência foi o local de instalação pós-alta para a maioria dos participantes. Os cuidadores dos pacientes eram seus próprios familiares (74/79 no D7 [75,5%]; 51/57 no D60 [89,4%]). Sobre orientações a respeito da LP, a primeira entrevista (D7) tomava como ponto de referência o momento da alta hospitalar. Dos entrevistados, 61/79 (77,2%) afirmaram ter recebido orientações no hospital.

Ressalta-se que a Atenção Primária foi o serviço de saúde que mais prestou assistência no D7, com 29/79 dos casos (36,7%). Houve uma proporção de pacientes sem assistência relacionada ao cuidado da LP desde as primeiras entrevistas, com 40/79 (50,6%) no D7 e 20/57 (35,1%) pacientes no D60.

Quanto à mobilidade, no D7, 40/79 (50,6%) pacientes foram classificados como acamados. No entanto, houve melhora progressiva da mobilidade, com as classificações “discreta limitação” e “sem limitação” aumentando 50% no último momento das entrevistas. Porém, no D60, 29/57 (50,9%) ainda estavam acamados ou com mobilidade muito limitada. A medida de alívio de pressão mais adotada foi o reposicionamento em 77/79 (97,5%) pacientes no D7 e em 46/57 pacientes (80,7%) no D60. O hidrogel e a papaína foram os curativos mais utilizados por 37/79 (46,8%) no D7. 

De acordo com o relato do paciente e/ou cuidador, no D60, foi percebida uma melhora da LP ou cicatrização em 48/57 pacientes (84,2%). 

### Fatores relacionados à evolução do processo cicatricial das LP

A fim de relacionar qual fator influenciou de maneira significativa o processo de cicatrização, foi analisada a resposta da última entrevista (D60) sobre o desfecho da cicatrização da LP. Os participantes foram divididos em dois grupos: Grupo 1, composto pelos que mencionaram cicatrização total ou melhora e Grupo 2, formado por aqueles que relataram estabilização ou piora da LP. Na Tabela 5 são apresentadas as variáveis que influenciam ou não na evolução do processo cicatricial.

**Tabela 5:** Variáveis analisadas para avaliar a relação com a evolução do processo cicatricial após 60 dias de acompanhamento. Botucatu, SP, Brasil, 2022-2023.

Variável	Melhora/ Cicatrização	Estabilizada/ Piora	HR* (IC 95%)	Valor p^†^
	n (%)*	n (%)*		
**Sexo**			
Feminino	26 (49)	24 (40)	1,1 (0,8-1,4)	0,385
Masculino	27 (51)	36 (60)	-	
**Cor**				
Brancos	39 (74)	46 (77)	1,0 (0,7-1,3)	0,876
Não brancos	14 (26)	14 (23)	-	
**Idade**				
≥ 65 anos	26 (49)	33 (55)	1,2 (0,7-2,0)	0,448
< 65 anos	27 (51)	27 (45)	-	
**Tempo de internação**				
≥ 30 dias	31 (59)	37 (62)	0,8 (0,5-1,5)	0,383
< 30 dias	22 (41)	23 (38)	-	
**Tempo para avaliação da comissão de curativos**				
≥ 15 dias	17 (32)	23 (38)	1,0 (0,5-1,7)	0,881
< 15 dias	36 (68)	37 (62)	-	
**Quantidade de LP^‡^**				
≥ 2	27 (51)	31 (52)	1,1 (0,8-1,4)	0,888
< 2	26 (49)	29 (48)	-	
**Hipertensão arterial**				
Sim	37 (70)	37 (62)	1,3 (0,7-2,3)	0,244
Não	16 (30)	23 (38)	-	
**Diabetes mellitus**				
Sim	13 (24)	20 (33)	1,2 (0,8-1,6)	0,202
Não	40 (76)	40 (67)	-	
**Acidente vascular encefálico**				
Sim	13 (24)	9 (15)	0,6 (0,3-1,0)	0,021
Não	40 (76)	51 (85)	-	

*HR = *Hazard Ratio;* ^†^Teste *Log Rank*; ^‡^LP = Lesão por pressão

Verificou-se que o fator que teve influência significativa no processo de cicatrização foi a presença de AVE (HR = 0,6; IC 95% 0,3-1,0; p = 0,021). Dos pacientes em que houve cicatrização dentro de 30 dias após a alta, 80% não sofreram AVE. A ausência dessa comorbidade foi um fator que influenciou a ter o desfecho de cicatrização mais precocemente. Entretanto, com o tempo de seguimento próximo ao D60, os pacientes com AVE atingiram resultados semelhantes aos pacientes que não tiveram AVE, evidenciando que essa comorbidade influenciou negativamente mais nas fases iniciais do processo de cicatrização.

## Discussão

O presente estudo acompanhou por 60 dias após a alta os pacientes que desenvolveram LP em ambiente hospitalar. Nesta coorte prospectiva, após a alta, muitos pacientes que desenvolveram LP tiveram desfechos desfavoráveis, pois a taxa de rehospitalização foi de 10,6% a 16,8% e a de óbito, 22,1%. Essas intercorrências denotam uma situação grave, a qual influencia a morbimortalidade dos pacientes que continuam em tratamento das LPs. Um estudo realizado nos Estados Unidos, com base em um extenso banco de dados americano, demonstrou que 25% dos casos de pacientes que desenvolveram LP hospitalar foram readmitidos nos 30 dias seguintes à alta e as taxas de readmissões mantiveram-se elevadas após 90 e 180 dias da alta hospitalar^23^.

No início das entrevistas, a maioria dos pacientes encontrava-se acamada. Contudo, observou-se uma melhora progressiva na mobilidade entre aqueles que não apresentaram intercorrências e participaram das entrevistas programadas, ao longo dos 60 dias de acompanhamento. Após a alta, 97,5% dos pacientes relataram a adoção do reposicionamento como estratégia para alívio de pressão, sendo reconhecida pela sua eficácia na redistribuição da pressão e fundamental, tanto para evitar a progressão de lesões quanto para promover a cicatrização^24^ ^-^ ^25^.

O AVE destacou-se como fator associado à progressão desfavorável do processo de cicatrização da LP. Pacientes acometidos por essa doença frequentemente apresentam prejuízo da mobilidade e redução de sensibilidade devido ao comprometimento neurológico^26^. Um estudo revelou que a prevalência de LP entre pacientes com AVE após a alta hospitalar foi significativamente maior do que quando estavam hospitalizados^27^, sugerindo que esse grupo de pacientes não recebeu cuidados nem atenção adequados para LP após a alta hospitalar. Quanto à localização anatômica das lesões, a região sacral foi a mais afetada, seguida pelos calcâneos - resultado semelhante aos outros estudos^28^ ^-^ ^29^, o que pode ser atribuído à predominância do decúbito dorsal durante a hospitalização. 

A alta hospitalar é um momento delicado e desafiador para os pacientes, os quais muitas vezes necessitam receber orientação e apoio para enfrentar as transições de cuidados^30^ ^-^ ^31^, especialmente aqueles com LP. É fundamental oferecer orientações sobre os cuidados de saúde no momento da alta hospitalar^32^ - no presente estudo, isso ocorreu com 61 dos 79 pacientes (77,2%). No entanto, uma investigação evidenciou que a maioria dos pacientes ou cuidadores não teve a oportunidade de praticar a técnica de realização de curativos durante o período de internação. Essa lacuna no treinamento prático pode resultar em dúvidas subsequentes, aumentando, assim, a probabilidade de erros e contribuindo para a ocorrência de readmissões hospitalares^33^. 

No Brasil, a Atenção Primária à Saúde (APS) é o primeiro nível de atenção do Sistema Único de Saúde (SUS), configurando-se como a principal porta de entrada dos usuários na rede assistencial^34^ e garantindo a continuidade do tratamento após a alta hospitalar. Estratégias como consultas presenciais, visitas domiciliares e telemonitoramento têm demonstrado impacto positivo na redução das taxas de reinternação, fortalecimento do vínculo entre usuário e equipe de saúde e maior adesão ao plano terapêutico^35^. Por outro lado, o acompanhamento tardio ou inexistente após a alta pode comprometer a efetividade da transição do cuidado, aumentar o risco de eventos adversos e favorecer novas internações, fragilizando o processo de recuperação do paciente^36^. Um exemplo é a quantidade de pacientes do presente estudo que ficou sem assistência, chegando a 50,6% na primeira semana. Portanto, é necessário aprimorar o processo de contrarreferência e a integração entre os serviços de saúde^37^, para que a responsabilidade de passar as informações não recaia apenas sobre o paciente.

A visita domiciliar, além de ampliar o cuidado com base na comunidade^38^, contribui para o atendimento individualizado daqueles que têm dificuldade de deslocamento até uma unidade de saúde, como é o caso da maioria dos pacientes com LP. Foi observado que, na entrevista D7, 29 pacientes (36,7%) receberam acompanhamento pela APS, sendo atendidos por meio de visitas domiciliares. 

Apesar de alguns participantes apresentarem desfechos favoráveis como melhora (29/57; 50,8%) e cicatrização total (19/57; 33,3%) das LPs, nem todos alcançaram o mesmo sucesso durante o período de acompanhamento, principalmente devido ao óbito ou reinternação. Pacientes que convivem com LP experimentam impactos negativos consideráveis, incluindo perda de independência, isolamento social, insatisfação e crença na falha da cura, além de diversos efeitos psicológicos que também afetam seus cuidadores^38^. 

Independentemente da complexidade da lesão, pacientes e seus cuidadores enfrentam desafios no gerenciamento das LP fora do ambiente hospitalar. Assim, a educação e a orientação adequada durante a alta hospitalar são essenciais e devem incluir instruções sobre as técnicas de troca de curativos, reposicionamento, coxins de alívio de pressão, entre outras medidas. Isso visa proporcionar aos pacientes e aos cuidadores o conhecimento e as habilidades necessárias para realizar o tratamento das LP eficazmente. Informações claras podem facilitar a compreensão dos pacientes e assegurar que os cuidadores estejam aptos a seguir os procedimentos adequados para a continuidade dos cuidados domiciliares após a alta^39^.

As falhas no planejamento dos cuidados durante a alta, caracterizadas pela fragmentação dos serviços, estão associadas aos desfechos clínico negativos, como o aumento na mortalidade, custos elevados de tratamento, ocorrência de erros médicos e readmissões não planejadas^33^. Reduzir a readmissão hospitalar é crucial não apenas pelos impactos desfavoráveis ao bem-estar do paciente, mas também pelas implicações financeiras ao sistema de saúde^39^.

A maioria dos participantes contou com algum membro da própria família como principal cuidador; no entanto seis indivíduos da amostra (8,6%) residiam em ILPI. Estudos indicam que as taxas de LP entre idosos institucionalizados são semelhantes àquelas observadas em contextos hospitalares^40^. Sabe-se que a maior parte dos idosos residentes em ILPI apresenta dependência funcional, especialmente no tocante à mobilidade, exigindo auxílio contínuo de profissionais de saúde. De maneira geral, todos os cuidadores, sejam familiares, sejam profissionais atuantes em ILPI, devem alinhar o cuidado pós-alta com o tratamento efetivo das LP^40^.

No decorrer do estudo, houve algumas limitações como as perdas de seguimento de pacientes, relacionadas às readmissões hospitalares frequentes e aos óbitos dos participantes. Além disso, houve dificuldades na realização das entrevistas telefônicas previstas para os diferentes momentos de coleta, o que comprometeu a obtenção completa dos dados ao longo do estudo. A não coleta de dados específicos sobre escolaridade e condições de moradia dos participantes também foi uma limitação, pois essas informações são importantes fatores sociais que influenciam os desfechos deste estudo. Isso é válido inclusive porque pacientes em situações socioeconômicas desfavoráveis tendem a apresentar maior vulnerabilidade ao desenvolvimento de LP^31^. Outra limitação refere-se à análise do processo cicatricial no período pós-alta, realizada com base na percepção de pacientes e/ou cuidadores e não por meio de métodos já validados. Entretanto, as informações coletadas referentes ao processo de cicatrização são apropriadas, uma vez que refletem a experiência do ponto de vista dos envolvidos. Apesar dessas limitações, o tamanho amostral final foi considerado adequado para a análise proposta, não comprometendo a validade da amostra. Todavia, estudos posteriores, com amostras mais robustas devem efetuar a análise multivariada dos fatores que podem influenciar a evolução do processo de cicatrização das LP hospitalares no período pós-alta.

O estudo demonstrou a realidade tanto do desenvolvimento de LP nos pacientes em ambiente intra-hospitalar quanto da evolução e cuidados prestados no período pós-alta. Esse conhecimento reforça a necessidade de estratégias capazes de melhorar os cuidados de prevenção dos pacientes de risco em ambiente hospitalar e contribuir na qualidade de vida do paciente após a alta. 

Recomenda-se a implementação de um plano de alta hospitalar estruturado para pacientes que desenvolveram LP, utilizando linguagem clara e acessível, com ilustrações e instruções detalhadas sobre a realização dos curativos e medidas de alívio da pressão. É essencial melhorar os mecanismos de comunicação entre as equipes de saúde para alinhar os cuidados, independentemente do nível de atenção ao qual o paciente será transferido^19^ ^,^ ^33^.

## Conclusão

Durante os 60 dias de seguimento pós-alta dos pacientes com LP hospitalar, cerca de 22% evoluíram para óbito. As taxas de reinternação variaram entre 10,6% e 16,8% ao longo do período. Observou-se que muitos pacientes não receberam assistência adequada dos serviços de saúde relacionada ao cuidado com a LP. Por outro lado, entre aqueles que foram acompanhados até o D60, houve melhora ou cicatrização da LP na grande maioria. Destaca-se que pacientes com AVE tiveram evolução mais lenta do processo de cicatrização.

Nossos achados sinalizam a urgência de melhorar as intervenções e o acompanhamento pós-alta, a fim de otimizar os processos de recuperação e cicatrização, com participação ativa do paciente, familiar e/ou cuidador. Identificar pacientes de maior risco, como aqueles que sofreram um AVE, e aprimorar as estratégias preventivas são medidas cruciais para elevar a qualidade do atendimento e dos desfechos aos pacientes com LP. Assim, ações de educação em saúde, construção de soluções inovadoras e avanços nos cuidados prestados contribuem para a eficácia do gerenciamento e da cicatrização de LP no ambiente domiciliar.

## Data Availability

Todos os dados gerados ou analisados durante este estudo estão incluídos neste artigo publicado.

# Translation: Evaluación post-alta de pacientes con lesión por presión hospitalaria: estudio de cohorte prospectivo^*^

**DOI:** 10.1590/1518-8345.7866.4767

**Authors:** Michelle Venâncio Hong, Bruna Cristina Velozo, Isabela Novello, Hélio Amante Miot, Meire Cristina Novelli e Castro, Luciana Patrícia Fernandes Abbade

**Affiliations:** 1 Universidade Estadual Paulista Júlio de Mesquita Filho, Faculdade de Medicina de Botucatu, Departamento de Enfermagem, Botucatu, SP, Brasil.; 2 Universidade Estadual Paulista Júlio de Mesquita Filho, Faculdade de Medicina de Botucatu, Departamento de Dermatologia, Botucatu, SP, Brasil.

**Keywords:** Úlcera por Presión, Alta del Paciente, Atención al Paciente, Servicios de Atención de Salud a Domicilio, Continuidad de la Atención al Paciente, Cicatrización de Heridas.

## Abstract

**Objetivo:**

evaluar la evolución cicatricial de las lesiones por presión desarrolladas en el entorno hospitalario de pacientes adultos en el período post-alta, así como las complicaciones y la atención brindada a estos pacientes.

**Método:**

estudio de cohorte prospectivo de pacientes que desarrollaron lesiones por presión durante la hospitalización y fueron dados de alta. Los datos se recolectaron a los 7, 15, 30 y 60 días después del alta hospitalaria.

**Resultados:**

la muestra incluyó a 113 pacientes, con 63 hombres (55,8%) y una edad promedio de 64,6 años (± 15,1). Hubo 246 lesiones, predominantemente en la región sacra (91; 37,0%) y estadio 2 (117; 47,6%). Las tasas de reingreso variaron del 10,6% al 16,8% durante 60 días y hubo 25 (22,1%) muertes. En la última entrevista, participaron 57 pacientes, de los cuales 29 (50,9%) permanecieron encamados o presentaron movilidad muy limitada y 48 (84,2%) presentaron mejoría o cicatrización de la lesión. Se observó ACV en 22 (19,5%) pacientes, lo que resultó ser un factor desfavorable para la cicatrización (HR = 0,6; p=0,021).

**Conclusión:**

durante los 60 días posteriores al alta, los pacientes presentaron resultados desfavorables, como fallecimiento, reingreso hospitalario y falta de cicatrización de las úlceras por presión. Los pacientes con accidente cerebrovascular presentaron una progresión más lenta de la cicatrización.

## Destacados

**(1)** Tras el alta hospitalaria, el 22% de los pacientes evolucionaron a óbito. **(2)** Las tasas de readmisión oscilaron entre el 10,6% y el 16,8%. **(3)** La presencia de la comorbilidad accidente cerebrovascular afectó negativamente el proceso de cicatrización. **(4)** En el 60º día post-alta, el 50,9% de las lesiones por presión mejoraron y el 33,3% cicatrizaron por completo. **(5)** Tras el alta, el 50,6% de los pacientes no recibió asistencia durante la primera semana.

## Introducción

La lesión por presión (LPP) se caracteriza por el daño a la piel o a los tejidos subyacentes causado por la aplicación de fuerza externa sobre la piel en zonas de prominencia ósea o por el uso de dispositivos médicos u otros objetos. Frecuente en hospitales, la LPP se considera un evento adverso y un indicador de la calidad de la atención prestada en los servicios de salud^1^.

A pesar de los avances en las estrategias de prevención, la incidencia de LPP se ha mantenido relativamente estable en los últimos años^2^. En lo que se refiere a LPP adquiridas por adultos hospitalizados, se estima que a nivel global que la prevalencia es del 12,8% y la incidencia, del 8,5%^3^. En Brasil, se notificaron más de 60.000 casos en 2023^4^. Existen variaciones significativas en la incidencia entre los diferentes sectores hospitalarios, siendo las Unidades de Cuidados Intensivos (UCI) las que presentan las tasas de prevalencia más altas (14,3%)^5^ ^-^ ^6^.

La LPP puede ocurrir debido a factores específicos del paciente, como edad avanzada, fragilidad cutánea^7^, movilidad reducida, cizallamiento, hospitalizaciones prolongadas^8^ ^)^ y desnutrición, lo cual constituye un factor de riesgo relevante^9^. Además de representar un desafío constante para la atención de la salud, la LPP es uno de los problemas clínicos más difíciles de manejar. Afecta negativamente a los pacientes a nivel emocional, mental, físico y social, lo que resulta en una reducción general de su calidad de vida^10^.

La LPP a menudo aumenta la dependencia de los sistemas de salud y las redes de apoyo^10^, lo que resulta en un aumento de la demanda de atención tanto durante la hospitalización como durante la transición al alta. Al momento del alta, existe una vulnerabilidad relacionada con la seguridad del paciente y su familia debido a la posible inconsistencia en información importante para la curación, como la atención tópica, las medidas de alivio de la presión y la evaluación frecuente de la piel^11^.

Los cambios ambientales resultantes de la hospitalización impactan no solo al paciente, sino también a sus familiares y cuidadores. Regresar a casa con una LPP no solo implica cambios en la rutina diaria, sino que también añade responsabilidades adicionales en el entorno doméstico^12^. Se recomiendan dispositivos de apoyo, mobiliario apropiado^13^, un espacio físico organizado para la higiene y la movilidad segura, y medidas de prevención de caídas^14^. Estos cambios, combinados con el apoyo del cuidador, garantizan una atención domiciliaria eficaz y segura. Sin embargo, a menudo no se abordan durante la hospitalización y el alta, lo que puede comprometer la atención de los pacientes con LPP y dificultar la cicatrización^15^.

Se sabe que es necesaria una orientación adecuada para familiares y cuidadores sobre el tratamiento y la prevención de la LPP. En este sentido, la estandarización de *las* guías post-alta permite reducir las lagunas de conocimiento sobre el tema^16^. Además, la atención de pacientes con LPP es extremadamente compleja, especialmente para personas sin formación profesional, lo que conlleva resultados desfavorables como reingresos hospitalarios, complicaciones y deterioro de la calidad de vida^15^.

A pesar de la relevancia del tema, faltan estudios que aborden la evolución tras el alta hospitalaria de pacientes con lesiones por presión adquiridas en entornos sanitarios y cómo los familiares o cuidadores informales monitorean estas lesiones en su domicilio. Por lo tanto, es importante investigar los resultados de las LPP en pacientes externados del ambiente hospitalario, así como las características del desarrollo de estos pacientes en el ámbito extrahospitalario.

Por ello, el objetivo de este estudio fue evaluar la evolución cicatricial de las LPP desarrolladas en el entorno hospitalario de pacientes adultos en el periodo post-alta, así como las complicaciones y la atención brindada a estos pacientes. 

## Método

### Tipo de estudio

Se trata de un estudio de cohorte prospectivo, informado de acuerdo con las directrices *Strengthening the Reporting of Observational studies in Epidemiology* (STROBE)^17^.

### Participantes radicación del estudio

El estudio incluyó a pacientes adultos que desarrollaron LPP durante su hospitalización en salas o UCI de dos hospitales públicos universitarios ubicados en la ciudad de Botucatu, en el interior de São Paulo, Brasil. Ambos hospitales pertenecen a la misma administración: uno es de nivel secundario y el otro de nivel terciario.

Las instituciones comparten el mismo Comité de Curaciones, compuesto por enfermeras especializadas que asisten a ambos hospitales en la atención de pacientes con lesiones por presión u otras lesiones que requieren cuidados específicos. Los pacientes con lesiones por presión, monitoreados durante la hospitalización y dados de alta, reciben orientación del Comité de Curaciones y de enfermeros especializados para un alta segura. Se proporciona una carta de contrarreferencia para el seguimiento en Atención Primaria, que incluye instrucciones y recomendaciones sobre las curaciones.

El estudio consideró pacientes evaluados por el Comité de Curaciones y que recibieron alta hospitalaria entre enero de 2022 y enero de 2023.

### Criterios de elegibilidad

Los criterios de inclusión para el estudio fueron: pacientes que desarrollaron LPP adquirida en el hospital, de ambos sexos, con una edad superior a 18 años, que recibieron evaluación por el Comité de Curaciones de la institución durante la estancia hospitalaria y que recibieron el alta hospitalaria con LPP.

Se excluyeron los que ya presentaban LPP al momento de su ingreso al hospital, incluso si desarrollaron nuevas lesiones durante la hospitalización y los pacientes con LPP que fueron transferidos a instituciones no incluidas en el estudio.

### Desenlace

El desenlace principal fue la evaluación de los factores asociados con la progresión del proceso de cicatrización dentro de los 60 días posteriores al alta hospitalaria. Con base en las percepciones de los cuidadores, los participantes se dividieron en dos grupos: progresión favorable de la cicatrización (cicatrización completa o mejoría) y progresión desfavorable de la cicatrización (estable o empeoramiento). Se asignaron los siguientes criterios en las entrevistas realizadas con pacientes, familiares o cuidadores: (i) Signos de mejoría: presencia de una herida con tejido rojizo (tejido de granulación), disminución del exudado y reducción del área y (ii) Signos de empeoramiento: aumento de la necrosis, olor, dolor, exudado y área sin cambios o aumentada.

Los resultados secundarios fueron: (i) Describir las características sociodemográficas, clínicas y terapéuticas basales de los pacientes que desarrollaron LPP en un entorno hospitalario; (ii) Relatar las complicaciones (muerte y readmisión) y las variables relacionadas con el cuidado del paciente durante el período de seguimiento post-alta, que se obtuvieron en las entrevistas.

### Instrumentos de recolección de datos

Para la identificación activa de pacientes, se realizó una búsqueda en los informes del Comité de Curaciones del sistema hospitalario. Estos informes verificaron si la LPP se produjo en el domicilio o en el hospital, seleccionando a todos los pacientes que desarrollaron LPP intrahospitalaria.

Para evaluar el cumplimiento de los criterios de elegibilidad, se accedió a la historia clínica electrónica a fin de obtener información del paciente. Tras la verificación y el consentimiento para participar mediante la obtención del Formulario de Consentimiento Libre e Informado (FCLI), se iniciaron entrevistas telefónicas con pacientes, familiares o cuidadores. Durante las entrevistas, se obtuvo información sobre las instrucciones de alta y su adherencia, los apósitos utilizados, el acceso a los servicios de salud, el estado clínico general y la evolución de las LPP.

### Recolección de datos y variables de estudio

La recopilación de datos se refirió al período de hospitalización (variables recopiladas a través de la historia clínica electrónica) y a los días 7 (D7), 15 (D15), 30 (D30) y 60 (D60) posteriores al alta hospitalaria (variables recopiladas mediante entrevistas). El período máximo de seguimiento de 60 días se estableció considerando la viabilidad de recopilar la información necesaria en este intervalo para evaluar la atención extrahospitalaria y la evolución del proceso de curación. Esta definición se basó en un estudio de cohorte retrospectivo que identificó un tiempo medio de 46 días para la cicatrización de la LP^18^.

Si el participante o su cuidador no contestaba el teléfono después de tres intentos, la entrevista del día se daba por no realizada. Sin embargo, el participante permanecía en el estudio y participaba en las entrevistas posteriores hasta completarlas. Al tratarse de un estudio observacional, no hubo interferencias en la atención rutinaria de los pacientes ni en las instrucciones para el alta.

Para recopilar datos, se utilizó un formulario en línea (Google Forms^®^) creado por los investigadores y validado tras una prueba piloto con diez pacientes. El formulario final incluyó variables dependientes e independientes del estudio, como:

- Datos sociodemográficos: sexo, edad, color/raza declarada (blanca, negra, mestiza, amarilla y/u otro) e ingresos familiares;
- Variables clínicas: comorbilidades relevantes, tiempo de estancia hospitalaria (en días), procedencia del paciente antes de su hospitalización, motivo de hospitalización, complicaciones durante la hospitalización y tiempo de estancia hospitalaria (en días) hasta la citación del Comité de Curaciones para evaluar la LP;
- Número de LPP desarrollados por paciente, localización anatómica y clasificación;
- Abordaje terapéutico de la LPP en el ámbito hospitalario.

- Entrevista posterior al alta: complicaciones (muerte y readmisión); lugar donde se instala el paciente después del alta (residencia propia, residencia de un familiar y/o amigos y Establecimientos de Larga Estancia para Adultos Mayores - ELEAM); cuidadores (familiar, amigo, técnico/auxiliar de enfermería o cuidador capacitado sin capacitación); si se brindó orientación después del alta hospitalaria; adherencia a las orientaciones posteriores al alta (se interrogó sobre la adherencia total o parcial a las orientaciones, o si no hubo adherencia); lugar de atención de salud después del alta; tipo y frecuencia de atención; movilidad que presentó el paciente al momento de la entrevista según la dimensión “movilidad” de la Escala de Braden^(19)^; medidas adoptadas para el tratamiento post-alta de las LPP; apósito utilizado al momento de la entrevista y evolución post-alta del proceso de cicatrización de la LPP, a partir de la perspectiva del paciente/cuidador (cicatrización total, mejoría, estabilización o empeoramiento).

### Definición de la muestra

Según datos anteriores del Comité de Curaciones de los hospitales incluidos en el estudio, se produjeron 269 LPP relacionadas con el hospital entre octubre de 2019 y octubre de 2020. El tamaño de muestra determinado para el estudio fue por conveniencia, considerando la recopilación de datos durante 12 meses para la inclusión de los participantes en el estudio.

### Análisis de datos

Se realizó estadística descriptiva de los datos mediante frecuencia y porcentaje para las variables categóricas, y mediante media, desviación estándar, mediana, mínimo y máximo para las variables cuantitativas. La normalidad de las distribuciones de las variables cuantitativas (continuas) se evaluó mediante la prueba de Shapiro-Wilk y los resultados de estas variables se presentaron como medias (± desviación estándar) cuando la distribución era normal y como mediana (p25 - p75) cuando la distribución no era normal^20^.

Se separaron dos grupos para el análisis: el grupo con evolución favorable (curación completa y mejoría del LP) y el grupo con evolución desfavorable (LPP estable o empeoramiento). Se realizó un análisis de la curva de supervivencia (Kaplan-Meier)^21^ ^)^ mediante la prueba *Log Rank* (Mantel-Cox) para identificar los factores que influyeron en la evolución favorable (mejoría/curación). En los casos con una diferencia en la supervivencia, el tamaño del efecto se estimó mediante el índice de riesgo *Hazard Ratio* (HR) y su intervalo de confianza del 95% (IC 95%)^22^. Los casos que no alcanzaron el resultado de evolución favorable fueron censurados porque abandonaron el estudio por pérdida de seguimiento (fallecimiento, readmisión o no completar la entrevista).

Las variables analizadas fueron escogidas según sus posibles influencias en el proceso de cicatrización y dicotomizadas según sus frecuencias medias, tales como sexo, edad mayor de 65 años, color/raza blanca versus no blanca, tiempo de hospitalización de 30 días, tiempo mínimo de 15 días para evaluación por el Comité de Curaciones, número de hasta dos LPP y comorbilidades [hipertensión arterial (HA), diabetes *mellitus* (DM) y accidente cerebrovascular (ACV)].

Todos los análisis estadísticos se realizaron con el programa SPSS 23.0 (IBM). Se consideró un intervalo de confianza (IC) del 95% y un nivel de significación del 5% (p < 0,05).

### Aspectos éticos

Este estudio fue aprobado por el Comité de Ética en Investigación y reconocido por la Comisión Nacional de Ética en Investigación con el Certificado de Presentación de Apreciación Ética n.º 53331521.6.0000.5411, cumpliendo con los estándares de las Resoluciones n.º 466/2012 y n.º 510/2016 del Consejo Nacional de Salud. Tras la aclaración y orientación sobre la investigación, la participación estuvo condicionada al consentimiento previo del paciente o su cuidador más cercano. Cuando el paciente no se encontraba en las instituciones debido al alta hospitalaria, el FCLI se obtuvo de forma remota.

## Resultados

Durante el período de estudio, el informe del sistema hospitalario registró 962 evaluaciones con el diagnóstico de “Lesión por Presión Hospitalaria” realizadas por el Comité de Curaciones. Tras eliminar los duplicados, es decir, los pacientes readmitidos en diferentes momentos, se excluyó a un total de 461 pacientes. Fueron excluidos los pacientes que no cumplieron los criterios de elegibilidad, es decir, aquellos que fallecieron en el hospital (n = 248), los menores de 18 años (n = 27), los transferidos a otra unidad hospitalaria (n = 9), los que presentaron una LPP al momento del ingreso hospitalario u otro tipo de lesión (n = 35) y aquellos para quienes no se solicitó interconsulta con el Comité de Curaciones (n = 9). Por lo tanto, 133 pacientes fueron elegibles, pero 113 dieron su consentimiento para participar y se unieron al estudio.

### Características sociodemográficas, clínicas y terapéuticas de los pacientes que desarrollaron LPP en el ámbito hospitalario

La Tabla 1 presenta los datos sociodemográficos y clínicos de los 113 participantes. La mayoría eran del sexo masculino (63; 55,8%), con edad promedio de 64,6 (± 15,1) años, color de piel/raza blanca autodeclarada (85; 75,2%) e ingresos familiares que oscilaban entre uno y tres salarios mínimos (49; 43,3%). La principal comorbilidad fue la hipertensión arterial (74; 65,5%). La duración de la estancia hospitalaria osciló entre 7 y 148 días, con una mediana de 40,0 (22,0 - 57,0). Antes de la hospitalización, 63 (55,8%) se encontraban en su domicilio, y los principales motivos de hospitalización estaban relacionados con lesiones por causas externas (25; 22,1%) y enfermedades del sistema nervioso (24; 21,2%). Las infecciones (52; 46,0%) fueron las complicaciones más observadas.

**Tabla 1:** Datos sociodemográficos y clínicos de los participantes con lesión por presión intrahospitalaria (n = 113). Botucatu, SP, Brasil, 2022-2023

**Variables**	**n (%)**
**Sexo**		
Masculino	63 (55,8)
Femenino	50 (44,2)
**Edad en años**		
Mínimo y máximo	22 a 95
Media (± desviación estándar)	64,6 (15,1)
**Color/raza**		
Blanca	85 (72,2)
Negra	17 (15,0)
Mestiza	11 (9,7)
**Ingresos familiares**		
Menos de 1 SM*	9 (7,9)
De 1 a 3 SM*	49 (43,3)
De 4 a 10 SM*	21 (18,5)
No informado	34 (30)
**Comorbilidades^†^**		
Hipertensión	74 (65,5)
Diabetes mellitus	34 (30,1)
Accidente cerebrovascular	22 (19,5)
**Periodo de hospitalización en días**		
Mínimo y máximo	7 a 148
Mediana (p25 - p75)	40,0 (22,0 - 57,0)
**Procedencia antes de la hospitalización**		
Domicilio propio	74 (65,5)
Transferido de otra hospitalización	32 (28,3)
Establecimientos de Larga Estancia para Adultos Mayores	5 (4,4)
Sin respuesta	2 (1,8)
**Motivo de hospitalización^†^**		
Lesiones por causas externas^‡^	25 (22,1)
Enfermedad del sistema nervioso	24 (21,2)
Enfermedad del tracto respiratorio	21 (18,6)
Enfermedad del sistema circulatorio	19 (16,8)
Neoplasias	11 (9,7)
Enfermedades endocrinas, metabólicas y nutricionales	10 (8,8)
Enfermedad del sistema genitourinario	10 (8,8)
Enfermedad del sistema digestivo	8 (7,1)
Enfermedad autoinmune	2 (1,8)
**Complicaciones durante la hospitalización^†^**		
Infecciones	52 (46,0)
Complicaciones respiratorias	43 (38,1)
Paro cardiopulmonar	10 (8,8)
Complicaciones cardiovasculares	10 (8,8)
Prueba de COVID-19 positiva	10 (8,8)
Trombosis venosa profunda	3 (2,7)
Accidente cerebrovascular	2 (1,8)
Infarto agudo de miocardio	1 (0,9)
Sin complicaciones	10 (8,8)

*SM = Salario mínimo vigente en 2022 (R$ 1.212,00), Brasil; ^†^Más de un resultado por cada paciente; ^‡^Lesiones por causas externas: fracturas, caídas, agresiones y accidentes relacionados con el transporte

Se contactó al Comité de Curaciones tras una mediana de 13,0 (6,0 - 29,0) días de hospitalización. Se identificaron 246 lesiones por presión en 113 pacientes, la mayoría de los cuales presentaban una sola lesión (58; 51,3%). La región sacra (91; 37,0%) y el calcáneo (49; 19,9%) fueron las localizaciones anatómicas más afectadas y la clasificación de lesión más identificada fue el estadio 2 (117; 47,6%) (Tabla 2).

La Tabla 3 muestra los enfoques terapéuticos y las medidas adoptadas para el alivio de la presión. El tratamiento tópico más comúnmente prescrito fue la polihexametilen biguanida (PHMB) en líquido o gel (109; 44,3%). La medida preventiva más recomendada fue el cambio de posición en la cama cada dos horas (173; 70,3%). Cabe destacar que el colchón antiescaras solo se prescribió a 38 participantes (15,4%).

**Tabla 2:** Características de las 246 lesiones por presión hospitalarias (n = 113). Botucatu, SP, Brasil, 2022-2023

**Variables**	**n (%)**
**Relacionadas con pacientes n = 113**		
**Duración de la estancia hospitalaria (en días) hasta que se contactó al Comité de Curaciones**		
Mínimo y máximo	1 a 105
Mediana (p25 - p75)	13 (6 - 29)
**Número de lesiones por presión desarrolladas por paciente**		
Uno	58 (51,3)
Tres	19 (16,8)
Más de tres	19 (16,8)
Dos	17 (15,0)
**Relacionadas con las lesiones por presión**	**n = 246**
**Ubicación anatómica**	
Sacro	91 (37,0)
Calcáneo	49 (19,9)
Isquiático	21 *(* 8,5)
Tobillo	17 (6,9)
Pabellón auricular	10 (4,1)
Trocánter	10 (4,1)
Pies o manos	8 (3,3)
Occipital	8 (3,3)
Regiones anatómicas*	32 (14,1)
**Clasificación de las lesiones por presión**		
Estadio 2	117 (47,6)
Estadio 1	60 (24,4)
Lesión inclasificable	31 (12,6)
Estadio 3	12 (4,9)
Lesión de tejido profundo	11 (4,5)
Lesión por dispositivo médico	11 (4,5)
Estadio 4	4 (1,6)

*Otras regiones anatómicas: maléolo lateral o medial, columna vertebral, escápula, tibial, nasal, rodilla, labios y codo

**Tabla 3:** Abordaje terapéutico tópico y guía para el alivio de la presión en 113 pacientes con 246 lesiones por presión intrahospitalarias. Botucatu, SP, Brasil, 2022-2023

**Variables**	**n (%)**
Tratamiento tópico en el ámbito hospitalario*		
Biguanida polihexametileno (PHMB) líquido o gel	109 (44,3)
Hidrogel	65 (26,4)
Protectores de piel^†^	89 (36,2)
Papaína	21 (8,5)
Placa de alginato de calcio	12 (4,9)
Tablero de espuma de poliuretano/hidropolímero	9 (3,7)
Colagenasa	8 (3,3)
Ácidos grasos esenciales	6 (2,4)
Hidrofibra con plata	6 (2,4)
Hidrocoloide	3 (1,2)
Sulfadiazina de plata	2 (0,8)
Apósito de presión negativa	1 (0,4)
Guías del Comité de Curaciones*		
Reposicionamiento en la cama cada dos horas	77 (68,1)
Almohadas/cojines	30 (26,5)
Hidratación de la piel y/o medidas preventivas	29 (25,6)
Colchón piramidal	27 (23,9)
Realizar cambio diario de apósitos	23 (20,3)
Alivio del talón	17 (15)
Protectores cutáneos^†^	17 (35,4)
Colchón antiescaras	14 (12,4)
Sin orientación	2 (1,7)

*Cada paciente podría tener más de un tratamiento o guía prescrita durante la hospitalización; ^†^Protectores de piel (crema barrera, film en aerosol y/o film de poliuretano)

### Resultados del periodo de seguimiento posterior al alta

Las variables de las entrevistas durante el período de 60 días después del alta hospitalaria se presentan en la Tabla 4. Durante este período, se constataron 25/113 (22,1%) óbitos. El número de reingresos varió entre entrevistas, pero en la primera semana después del alta, 19 (16,8%) pacientes regresaron al hospital y en D60, 12 (10,6%) regresaron. En el transcurso de las entrevistas, el número de pacientes/cuidadores entrevistados disminuyó debido a complicaciones (muerte, reingreso o no haber completado la entrevista por no responder a las llamadas telefónicas). Inicialmente, se entrevistó a 79 (69,9%), pero al final, hubo 57 (50,4%) participantes en la muestra total.

**Tabla 4:** Variables relacionadas con los 113 pacientes con lesiones por presión hospitalizados dentro de los 60 días posteriores al alta hospitalaria. Botucatu, SP, Brasil, 2022-2023

**Variables**	D7* n (%)	D15* n (%)	D30* n (%)	D60* n (%)
**No entrevistado - Razones**	34 (30)	38 (33,6)	36 (31,8)	36 (35,4)
Muerte^†^	5 (4,4)	10 (8,8)	15 (13,3)	25 (22,1)
Readmisión	19 (16,8)	17 (15,0)	19 (16,8)	12 (10,6)
No respondió al contacto telefónico	10 (8,8)	16 (14,2)	12 (10,6)	19 (16,8)
**Entrevistados**	79 (69,9)	70 (61,9)	67 (59,3)	57 (50,4)
**Ubicación de la instalación**			
Domicilio propio	46 (58,2)	41 (58,6)	43 (64,2)	36 (63,2)
Domicilio de familiares y/o amigos	28 (35,4)	23 (32,9)	19 (28,4)	18 (31,6)
Establecimientos de Larga Estancia para Adultos Mayores	5 (6,3)	6 (8,6)	5 (7,5)	3 (5,3)
**Cuidadores^‡^**				
Familia	74 (75,5)	64 (68,8)	61 (70,9)	51 (89,5)
Cuidador sin capacitación	13 (13,3)	16 (17,2)	16 (18,6)	8 (14)
Técnico/a de enfermería o asistente	9 (9,2)	11 (11,8)	8 (9,3)	4 (7,0)
Amigo	2 (2)	2 (2,2)	1 (1,2)	1 (1,8)
**Hubo orientación sobre las lesiones por presión**			
Sí	61 (77,2)	62 (88,6)	55 (82,1)	49 (86,0)
No	18 (22,8)	8 (11,4)	12 (17,9)	8 (14)
**Adherencia a las guías sobre lesiones por presión**			
Sí, adherencia total	57 (72,2)	59 (84,3)	54 (80,6)	48 (84,2)
Sí, adherencia parcial	4 (5,1)	4 (5,7)	1 (1,5)	0 (0,0)
No	18 (22,8)	7 (10,0)	12 (17,9)	9 (15,8)
**Asistencia de cuidados^‡^**			
Sin asistencia	40 (50,6)	23 (32,9)	23 (34,3)	20 (35,1)
Atención Primaria de Salud	29 (36,7)	28 (40,0)	20 (29,9)	19 (33,3)
Clínica Ambulatoria Especializada y/o Clínica Privada	7 (8,9)	23 (32,9)	25 (37,3)	19 (33,3)
Sala de Emergencia	4 (5,1)	EN	1 (1,5)	1 (1,8)
**Tipo de asistencia^‡^**				
Sin asistencia	40 (50,6)	23 (32,9)	23 (34,3)	20 (35,1)
Visita a domicilio	30 (38,0)	29 (41,4)	24 (35,8)	20 (35,1)
Atención en el propio centro de salud	9 (11,4)	19 (27,1)	22 (32,8)	18 (31,6)
**Frecuencia de asistencia**				
Sin asistencia	40 (50,6)	23 (32,9)	23 (34,3)	20 (35,1)
Semanal	32 (40,5)	26 (37,1)	21 (31,3)	16 (28,1)
Quincenal	-	21 (30,0)	15 (22,4)	5 (8,8)
Mensual	-	-	10 (14,9)	16 (28,1)
**Movilidad**				
Acamado	40 (50,6)	26 (37,1)	23 (34,3)	16 (28,1)
Muy limitado	25 (31,6)	28 (40)	25 (37,3)	13 (22,8)
Discreta limitación	9 (11,4)	13 (18,6)	11 (16,4)	18 (31,6)
Sin limitación	5 (6,3)	3 (4,3)	8 (11,9)	10 (17,5)
**Medidas de alivio de presión^‡^**				
Reposicionamiento	77 (97,5)	68 (97,1)	61 (91,0)	46 (80,7)
Almohadones	31 (39,2)	30 (42,9)	29 (43,3)	24 (42,1)
Colchones piramidales y neumáticos	17 (21,5)	23 (32,9)	21 (31,3)	10 (17,5)
**Curación utilizada^‡^**				
Crema barrera	37 (46,8)	28 (40,0)	28 (41,8)	18 (31,6)
Hidrogeles/papaínas	37 (46,8)	34 (48,6)	41 (58,6)	20 (35,1)
Ácido graso esencial	26 (32,9)	24 (34,3)	19 (28,4)	14 (24,6)
Sin utilización de producto	3 (3,8)	4 (5,7)	8 (11,9)	13 (22,8)
Otros^§^	12 (15,2)	14 (20,0)	7 (10,4)	14 (24,6)
**Evolución del proceso de cicatrización**				
Mejoría	53 (67,1)	54 (77,1)	49 (73,1)	29 (50,9)
Estabilización	20 (25,3)	8 (11,4)	3 (4,5)	6 (10,5)
Cicatrización total	5 (6,3)	6 (8,6)	11 (16,4)	19 (33,3)
Empeoramiento	1 (1,3)	2 (2,9)	4 (6)	3 (5,3)

*D7, D15, D30 y D60: Se refieren a los días de seguimiento después del alta hospitalaria, respectivamente 7º, 15º, 30º y 60º día; ^†^Muerte: a partir del D15, el número total de óbitos pasó a representar la suma acumulada de eventos ocurridos en visitas anteriores; ^‡^Más de un resultado para cada paciente; ^§^Otras curaciones: película en aerosol, colagenasa, placa de alginato de calcio y sodio y polihexametilen biguanida (PHMB) en líquido o gel

Para la mayoría de los participantes, el propio domicilio fue el lugar de instalación tras el alta. Los cuidadores de los pacientes fueron sus propios familiares (74/79 en el D7 [75,5%]; 51/57 en el D60 [89,4%]). En cuanto a las orientaciones sobre la LPP, la primera entrevista (D7) tomó como punto de referencia el momento del alta hospitalaria. De los entrevistados, 61/79 (77,2%) informaron haber recibido orientación en el hospital.

Cabe destacar que Atención Primaria fue el servicio de salud que más asistencia brindó el día 7, con 29/79 casos (36,7%). Hubo una proporción de pacientes sin asistencia relacionada con la atención de LPP desde las primeras entrevistas: 40/79 (50,6%) el día 7 y 20/57 (35,1%) el día 60.

En cuanto a la movilidad, el D7, 40/79 (50,6%) pacientes fueron clasificados como acamados. Sin embargo, se observó una mejora progresiva de la movilidad, con un aumento del 50% en las clasificaciones de “discreta limitación” y “sin limitación” en la última entrevista. No obstante, el D60, 29/57 (50,9%) seguían acamados o presentaban movilidad muy limitada. La medida de alivio de la presión más utilizada fue el reposicionamiento en 77/79 (97,5%) pacientes el D7 y en 46/57 (80,7%) el D60. El hidrogel y la papaína fueron los apósitos más utilizados por 37/79 (46,8%) pacientes el D7.

Según el informe del paciente y/o cuidador, el día 60 se observó una mejoría de la LPP o cicatrización en 48/57 pacientes (84,2%).

### Factores relacionados con la evolución del proceso de curación de las LPP

Para determinar qué factores influyeron significativamente en el proceso de curación, se analizaron las respuestas de la entrevista final (D60) sobre el resultado de la curación de la LPP. Los participantes se dividieron en dos grupos: el Grupo 1, compuesto por quienes reportaron curación completa o mejoría y el Grupo 2, compuesto por quienes reportaron estabilización o empeoramiento de la LPP. La Tabla 5 presenta las variables que influyen o no en la progresión del proceso de curación.

**Tabla 5:** Variables analizadas para evaluar su relación con la evolución del proceso de cicatrización tras 60 días de seguimiento. Botucatu, SP, Brasil, 2022-2023

Variable	Mejoría/ Cura	Estabilización/ Empeoramiento	HR* (IC del 95%)	valor p^†^
	n (%)*	n (%)*		
**Sexo**			
Femenino	26 (49)	24 (40)	1,1 (0,8-1,4)	0,385
Masculino	27 (51)	36 (60)	-	
**Color**				
Blancos	39 (74)	46 (77)	1,0 (0,7-1,3)	0,876
No blancos	14 (26)	14 (23)	-	
**Edad**				
≥ 65 años	26 (49)	33 (55)	1,2 (0,7-2,0)	0,448
< 65 años	27 (51)	27 (45)	-	
**Duración de la estancia hospitalaria**				
≥ 30 días	31 (59)	37 (62)	0,8 (0,5-1,5)	0,383
< 30 días	22 (41)	23 (38)	-	
**Evaluación por parte del Comité de Curaciones**				
≥ 15 días	17 (32)	23 (38)	1,0 (0,5-1,7)	0,881
< 15 días	36 (68)	37 (62)	-	
**Cantidad de LPP^‡^**				
≥ 2	27 (51)	31 (52)	1,1 (0,8-1,4)	0,888
< 2	26 (49)	29 (48)	-	
**Hipertensión arterial**				
Sí	37 (70)	37 (62)	1,3 (0,7-2,3)	0,244
No	16 (30)	23 (38)	-	
**Diabetes mellitus**				
Sí	13 (24)	20 (33)	1,2 (0,8-1,6)	0,202
No	40 (76)	40 (67)	-	
**Accidente cerebrovascular**				
Sí	13 (24)	9 (15)	0,6 (0,3-1,0)	0,021
No	40 (76)	51 (85)	-	

*HR = *Hazard Ratio*; ^†^Prueba de *Log Rank*; ^‡^LPP = Lesión por presión

El factor que influyó significativamente en el proceso de curación fue la presencia de ACV (HR = 0,6; IC del 95%: 0,3-1,0; p = 0,021). De los pacientes en que hubo cicatrización en los 30 días posteriores al alta, el 80% no sufrió ACV. La ausencia de esta comorbilidad influyó en la curación temprana. Sin embargo, con un seguimiento cercano al día 60, los pacientes con ACV obtuvieron resultados similares a los pacientes sin ACV, lo que demuestra que esta comorbilidad influyó negativamente en las fases iniciales del proceso de cicatrización.

## Discusión

El presente estudio realizó un seguimiento de pacientes que desarrollaron LPP en el entorno hospitalario durante 60 días tras el alta. En esta cohorte prospectiva, muchos pacientes que desarrollaron LPP tuvieron resultados desfavorables tras el alta, con tasas de reingreso que oscilaron entre el 10,6% y el 16,8% y tasas de mortalidad que alcanzaron el 22,1%. Estas complicaciones representan una situación grave que influye en la morbilidad y la mortalidad de los pacientes que continúan recibiendo tratamiento para LPP. Un estudio realizado en Estados Unidos, fundado en una extensa base de datos estadounidense, demostró que el 25% de los pacientes que desarrollaron LPP intrahospitalaria fueron readmitidos en los 30 días siguientes al alta y las tasas de reingreso se mantuvieron altas entre los 90 y los 180 días posteriores al alta hospitalaria^23^.

Al inicio de las entrevistas, la mayoría de los pacientes se encontraban en cama. Sin embargo, se observó una mejora progresiva de la movilidad en quienes no presentaron complicaciones y participaron en las entrevistas programadas, durante el período de seguimiento de 60 días. Tras el alta, el 97,5% de los pacientes refirió haber adoptado el reposicionamiento como estrategia de alivio de la presión, lo cual es reconocido por su eficacia para redistribuir la presión y es esencial, tanto para prevenir la progresión de la lesión, como para promover la cicatrización^24^ ^-^ ^25^.

El ACV se destacó como un factor asociado con la progresión desfavorable del proceso de curación de la LLP. Los pacientes con esta enfermedad a menudo presentan movilidad y sensibilidad reducidas debido al deterioro neurológico^26^. Un estudio reveló que la prevalencia de LPP entre los pacientes con accidente cerebrovascular después del alta hospitalaria fue significativamente mayor que durante la hospitalización^27^, lo que sugiere que este grupo de pacientes no recibió atención y cuidados adecuados para la LPP después del alta hospitalaria. En cuanto a la ubicación anatómica de las lesiones, la región sacra fue la más afectada, seguida del calcáneo, un resultado similar a otros estudios^28^ ^-^ ^29^, que puede atribuirse al predominio de la posición supina durante la hospitalización.

El alta hospitalaria es un momento delicado y desafiante para los pacientes, quienes a menudo requieren orientación y apoyo para afrontar las transiciones de atención^30^ ^-^ ^31^, especialmente aquellos con LPP. Brindar orientación sobre la atención médica al momento del alta hospitalaria es esencial^32^ - en el presente estudio, esto ocurrió con 61 de 79 pacientes (77,2%). Sin embargo, un estudio encontró que la mayoría de los pacientes o cuidadores no tuvieron la oportunidad de practicar técnicas de vendaje durante su hospitalización. Esta brecha en la capacitación práctica puede resultar en preguntas posteriores, lo que aumenta la probabilidad de errores y contribuye a los reingresos hospitalarios^33^.

En Brasil, la Atención Primaria de Salud (APS) es el primer nivel de atención dentro del Sistema Único de Salud (SUS), dado que opera como puerta principal de ingreso de pacientes a la red de atención médica^34^ ^)^ y asegura la continuidad del tratamiento después del alta hospitalaria. Estrategias como las consultas presenciales, las visitas domiciliarias y la telemonitorización han demostrado impactos positivos en la reducción de las tasas de readmisión, el fortalecimiento del vínculo entre los pacientes y los equipos de atención médica y el aumento de la adherencia a los planes de tratamiento^35^. Por el contrario, el seguimiento tardío o inexistente después del alta puede comprometer la efectividad de la transición de la atención, aumentar el riesgo de eventos adversos y favorecer las rehospitalizaciones, lo que debilita el proceso de recuperación del paciente^36^. Un ejemplo es el número de pacientes en este estudio que quedaron sin atención, llegando al 50,6% en la primera semana. Por lo tanto, es necesario mejorar el proceso de contrarreferencia y la integración entre los servicios de atención médica^37^ ^)^ para que la responsabilidad de brindar información no recaiga únicamente en el paciente.

Las visitas domiciliarias, además de ampliar la atención comunitaria^38^, contribuyen a la atención individualizada para quienes tienen dificultades para desplazarse. a una unidad de salud, como es el caso de la mayoría de los pacientes con LPP. Se observó que, en la entrevista D7, 29 pacientes (36,7%) recibieron seguimiento por parte de la APS, con atención mediante visitas domiciliarias.

Si bien algunos participantes presentaron resultados favorables, como mejoría (29/57; 50,8%) y curación completa (19/57; 33,3%) de las LPP, no todos lograron el mismo éxito durante el período de seguimiento, principalmente debido al fallecimiento o al reingreso. Los pacientes con LPP experimentan considerables impactos negativos, como pérdida de independencia, aislamiento social, insatisfacción y la creencia en el fracaso de la curación, además de diversos efectos psicológicos que también afectan a sus cuidadores^38^.

Independientemente de la complejidad de la lesión, los pacientes y sus cuidadores se enfrentan a desafíos en el manejo de las lesiones por presión fuera del ámbito hospitalario. Por lo tanto, es esencial brindar una educación y orientación adecuadas durante el alta hospitalaria, que incluyan instrucciones sobre técnicas de cambio de vendajes, reposicionamiento, almohadillas de alivio de presión y otras medidas. Esto busca brindar a los pacientes y cuidadores los conocimientos y las habilidades necesarias para manejar eficazmente las lesiones por presión. Una información clara facilita la comprensión del paciente y garantiza que los cuidadores puedan seguir los procedimientos adecuados para continuar la atención domiciliaria después del alta^39^.

Las fallas en la planificación de la atención durante el alta, caracterizadas por la fragmentación de los servicios, se asocian con resultados clínicos negativos, como aumento de la mortalidad, altos costos de tratamiento, errores médicos y reingresos no planificados^33^. Reducir los reingresos hospitalarios es crucial no solo por los impactos adversos en el bienestar del paciente, sino también por las implicaciones financieras para el sistema de salud^39^.

La mayoría de los participantes contó con algún miembro de su propia familia como cuidador principal; sin embargo, seis personas de la muestra (8,6%) residían en Establecimientos de Larga Estancia para Adultos Mayores (ELEAM). Los estudios indican que las tasas de LPP entre las personas mayores institucionalizadas son similares a las observadas en entornos hospitalarios^40^. Se sabe que la mayoría de las personas mayores que residen en ELEAM presentan dependencia funcional, especialmente en lo que respecta a la movilidad y requieren asistencia continua de profesionales de la salud. En general, todos los cuidadores, ya sean familiares o profesionales que prestan servicios en ELEAM, deben alinear la atención posterior al alta con el tratamiento eficaz de las LP^40^.

Durante el estudio, surgieron algunas limitaciones, como pérdidas de pacientes durante el seguimiento, relacionadas con frecuentes reingresos hospitalarios y muertes de participantes. Además, se produjeron dificultades en la realización de las entrevistas telefónicas programadas para los diferentes puntos de recolección, lo que comprometió la recopilación completa de datos a lo largo de la investigación. La falta de recopilación de datos específicos sobre la educación y las condiciones de vivienda de los participantes también fue una limitación, ya que estos datos son factores sociales importantes que influyen en los resultados de este estudio. Esto es así porque los pacientes en situaciones socioeconómicas desfavorables tienden a ser más vulnerables a desarrollar LPP^31^. Otra limitación se refiere al análisis del proceso de curación en el período posterior al alta, que se realizó con base en las percepciones de los pacientes y/o cuidadores, en lugar de utilizar métodos previamente validados. Sin embargo, la información recopilada sobre el proceso de cicatrización es apropiada, ya que refleja la experiencia desde la perspectiva de los implicados. A pesar de estas limitaciones, el tamaño final de la muestra se consideró adecuado para el análisis propuesto, sin comprometer la validez de la muestra. Sin embargo, estudios futuros con muestras más robustas deberían realizar análisis multivariados de los factores que pueden influir en la evolución del proceso de curación de las LPP hospitalarios en el periodo post-alta.

El estudio demostró la realidad tanto del desarrollo de la LPP en pacientes hospitalizados como de su progresión y atención tras el alta. Este conocimiento refuerza la necesidad de estrategias que mejoren la atención preventiva de los pacientes en riesgo en el hospital y contribuyan a su calidad de vida tras el alta.

Se recomienda implementar un plan de alta hospitalaria estructurado para pacientes que hayan desarrollado LPP, utilizando un lenguaje claro y accesible, con ilustraciones e instrucciones detalladas sobre cómo realizar los vendajes y las medidas de alivio de la presión. Es esencial mejorar los mecanismos de comunicación entre los equipos de salud para armonizar la atención, independientemente del nivel de atención al que se transfiera al paciente^19^ ^,^ ^33^.

## Conclusión

Durante el seguimiento de 60 días tras el alta de pacientes con LPP, aproximadamente el 22% evolucionó a óbito. Las tasas de reingreso oscilaron entre el 10,6% y el 16,8% durante todo el período. Se observó que muchos pacientes no recibieron la atención adecuada de los servicios de salud relacionados con la atención de LPP. Por otro lado, entre los pacientes con seguimiento hasta el día 60, la LPP mejoró o se curó en la gran mayoría. Cabe destacar que los pacientes con ACV presentaron una evolución más lenta en el proceso de cicatrización.

Nuestros hallazgos resaltan la urgente necesidad de mejorar las intervenciones y el seguimiento posterior al alta para optimizar los procesos de recuperación y curación, con la participación activa de pacientes, familias y/o cuidadores. Identificar a los pacientes de mayor riesgo, como aquellos que han sufrido un ACV y mejorar las estrategias preventivas son medidas cruciales para optimizar la calidad de la atención y los resultados de los pacientes con LPP. Por lo tanto, las iniciativas de educación sanitaria, el desarrollo de soluciones innovadoras y las mejoras en la atención contribuyen al manejo y la cicatrización eficaz de las LPP en el entorno domiciliario.

## Data Availability

Todos los datos generados o analizados durante este estudio están incluidos en este artículo publicado.
